# Epigenetic silencing of innate and adaptive immunity genes underlies the immune evasion cancer hallmark in *Theileria*-infected B-cells

**DOI:** 10.1371/journal.ppat.1014498

**Published:** 2026-08-28

**Authors:** Marisol Giacomini, Aristeidis Panagiotou, Steve G. Odette, Ariane Honfozo, Jeremy Berthelet, Angelique Amo, Joseph M. Dybas, Julia Orio-Tejada, Katarzyna Kulej, Marie Villares, Benjamin A. Garcia, Magali Hennion, Matthew D. Weitzman, Jonathan B. Weitzman

**Affiliations:** 1 Université Paris Cité, CNRS, UMR7126 Epigenetics and Cell Fate, Paris, France; 2 Department of Pathology and Laboratory Medicine, University of Pennsylvania Perelman School of Medicine, Philadelphia, Pennsylvania, United States of America; 3 Division of Cancer Pathobiology and Division of Protective Immunity, The Children’s Hospital of Philadelphia, Philadelphia, Pennsylvania, United States of America; 4 Department of Biochemistry and Molecular Biophysics, Washington University School of Medicine, St. Louis, Missouri, United States of America; 5 Institut Universitaire de France (IUF), Paris, France; Radboud University: Radboud Universiteit, NETHERLANDS, KINGDOM OF THE

## Abstract

Cancer hallmarks are characterized by wide-scale changes in gene expression programs. Pioneering studies showed how viral oncogenes target regulatory pathways, but little is known about tumorigenic mechanisms of non-viral pathogens. *Theileria annulata* is an intracellular parasite (related to apicomplexa parasites causing malaria) which remarkably transforms bovine leukocytes, hijacking host signaling pathways to induce cancer phenotypes, akin to human leukemias. While some host genes contribute to the proliferative or invasive hallmark phenotypes, there is still limited comprehensive understanding of the impact of *Theileria* infection on host transcription and transformation. We performed a multi-omics meta-analysis to investigate the effect of *Theileria* infection on cancer hallmarks in bovine B lymphocytes. Combining transcriptomic, proteomic and epigenomic analysis across multiple datasets, we show that *Theileria* infection suppresses host immune pathways. Specifically, genes encoding innate and adaptive immune mediators are repressed in *T. annulata*-infected B cells (and in *T. parva* infected T cells), including downregulation of genes for Toll-like receptors (TLR), inflammasome components of the guanylate binding protein (GBP) family and major histocompatibility complex class (MHC) II genes. Treatment with distinct theilericidal drugs could partially rescue immune gene expression. Mechanistically, we describe alterations in the host epigenome, including loss of activating histone modifications (e.g., H3K18ac, H3K4me3, H3K27ac) on the promoters of repressed immune genes, and enrichment of silencing marks (H3K27me3) on promoters of the *BOLA* genes and the gene encoding CIITA, the master transcriptional regulator of MHC class II gene expression. Our results suggest that intracellular *T*. *annulata* and *T. parva* parasites could drive an immune evasion cancer hallmark in host lymphocytes by epigenetic silencing of genes for innate and adaptive immunity.

## Author summary

Cancers and infectious diseases often exploit similar strategies to evade immune defenses. *Theileria* are tick-borne intracellular parasites that infect cattle. *Theileria annulata* parasites infect bovine B cells and macrophages and induce cancer-like traits, yet how they persist inside host cells remains unclear. We integrated transcriptomic, proteomic, and epigenomic analyses to discover that *Theileria* parasites broadly silence a range of host genes that are required for innate and adaptive immunity. These include Toll-like receptors, inflammasome components, and major histocompatibility complex class II molecules. This epigenetic repression could deregulate the interaction of infected cells with the immune system. Importantly, treatment with different anti-*Theileria* drugs reversed this silencing mechanism. Our findings reveal a parasite-driven immune evasion mechanism that mirrors cancer hallmarks and highlights potential therapeutic opportunities targeting host–parasite epigenetic interactions.

## Introduction

The realization that cancer cells share a set of well-characterized phenotypes, has fueled the intense search for mechanisms and genetic events that contribute to the establishment of each of these phenotypes. Since the famous description of the ‘Hallmarks of Cancer” by Hanahan and Weinberg [1,2], these are often used as an entry point to dissect specific tumor types in terms of the contributing mutations that lead to cancer hallmarks. Many studies have demonstrated how viral infections that cause cancer lead to specific targeted events that contribute to the set of cancer phenotypes [3–5]. However, the impact of non-viral pathogens (particularly intracellular parasites) on tumorigenesis and oncogenic regulatory pathways is relatively unexplored [6]. Here, we investigate the cancer hallmarks within the context of the transformation of bovine leukocytes by the intracellular parasite *Theileria annulata*, which induces a set of cancer-like phenotypes in the absence of genetic mutations.

The Apicomplexa phylum includes intracellular parasites such as *Plasmodium* and *Toxoplasma*, causing malaria and toxoplasmosis, respectively. *Theileria* spp. are unique among apicomplexa, as some *Theileria* species can transform host cells, providing a model for studying the impact of parasite-host interactions [7–10]. *T. annulata* is a tick-borne, pathogen that causes Tropical Theileriosis in cows (as well as infecting sheep and goats), resulting in 1.1 million cattle deaths annually with an economic impact of over $168 million [11]. *T. annulata* and *T. parva* species induce a lymphoproliferative disease in cows, akin to some human leukemias [12,13]. These *Theileria* species immortalize and transform host cells, driving proliferation, migration, and metastasis; and these features can be partially reversed by treatment with theilericidal drugs, such as Buparvaquone [13–17]. Parasite-induced leukocyte transformation provides a remarkable system for exploring signaling and epigenetic mechanisms in parasite-induced cellular phenotypes [9]. Indeed, a Cancer Hallmarks approach was proposed for considering *Theileria-*induced transformation [13]. There has been some success in identifying host gene regulation events linked to parasite-induced cancer phenotypes [17–19]. For example, B lymphocytes infected by *T. annulata* display elevated expression of the gene encoding Matrix Metalloprotease-9 (MMP-9) which contributes to the ‘*Invasion and metastasis*’ hallmark [20,21]. Furthermore, the secreted parasite protein TaPIN1 activates the expression of host c-Jun, a proto-oncogenic transcription factor that drives *MMP9* expression and host cell proliferation [22,23]. Other studies have provided clues into the ‘*Deregulation of cellular energetics*’ hallmark, demonstrating that lymphocytes infected with *T. annulata* display a Warburg-like effect that is associated with deregulated levels of Reactive oxygen species (ROS), activation of the Hypoxia-inducible factor 1α (HIF-1α) and induction of metabolic regulators genes, such as Hexokinase 2 (HK2) [24,25]. Despite these insights, the network of mechanisms that enable bovine leucocytes infected with *T. annulata* to behave like cancer cells remains relatively unclear [7,13]. While some studies have linked *Theileria* infection to specific transcription factors (such as AP-1 [26,27] or HIF1α [24,25], it is also emerging that epigenetic factors may contribute to parasite-induced transformation [7,9]. For example, upregulation of the SMYD3 methyltransferases accompanies parasite induction of the metastatic *MMP9* gene [21]. Indeed, beyond mutations, epigenetic rewiring likely contributes to the establishment and maintenance of cancer hallmarks [28]. Epigenetic mechanisms and enzymes represent promising drug targets for cancer therapies [29,30].

Previous studies on *Theileria* induced transformation have tended to start with a specific hypothesis about a given cancer-related pathway, subsequently zooming in on mechanistic details [10]. Here we describe a non-biased approach based on cancer hallmark analysis of transcriptome and proteome datasets across multiple cell lines.

This multi-omics meta-analysis led us to investigate the impact of infection by *T. annulata*, or related *T. parva* parasites, on the expression of innate and adaptive immune receptors, which could underlie the ‘*Immune evasion and Inflammation*’ hallmark that is essential for parasite-infected cells to escape the immune elimination in cows. The Bovine Leukocyte Antigen (BoLA or BOLA) locus is the cow equivalent of the human HLA or Major Histocompatibility Complex (MHC), which is critical for antigen presentation and immune signaling [31]. Many pathogens, notably viruses, evade host immune recognition by targeting HLA components via different mechanisms [32]. In addition to the antigen presentation machinery of the adaptive immune response, cells have innate immune responses that provide defence against invading pathogens. The innate immune response relies on the recognition of conserved features of pathogen-specific molecules. Innate immune defences include members of the Toll-like receptor (TLR) family that recognize pathogen-associated immunostimulants, including lipopolysaccharide (LPS), peptidoglycans or CpG DNA [33,34]. Other innate immune response machinery includes the inflammasomes, cytosolic molecular factories that sense stimuli associated with pathogen infection or cellular stress and lead to rapid induction of proinflammatory cytokines responses and apoptotic pathways [35]. Again, many pathogens have evolved sophisticated strategies to counteract the innate immune response systems to avoid immune recognition and immune destruction [36,37]. These have been well-studied in viral and bacterial infections, but less is known about how intracellular parasites survive in host cells and avoid recognition by the immune system [34].

Here, we describe a Cancer Hallmarks approach to explore the impact of infection by *T. annulata* and *T. parva* on the expression of components of the bovine innate and adaptive immune response. We analyzed transcriptome, proteome and epigenome datasets to hone in on the regulation of both innate and adaptive immune genes important for pathogen recognition and antigen presentation. We provide mechanistic insight by showing that the wide-scale repression of host *BOLA*, *TLR* and *GBP* genes is associated with epigenetic silencing of the MHC transcriptional regulator CIITA and changes in the epigenomic landscape.

## Results

### Meta-analysis of *Theileria*-induced transcriptomics highlights cancer hallmarks

**Fig 1:**
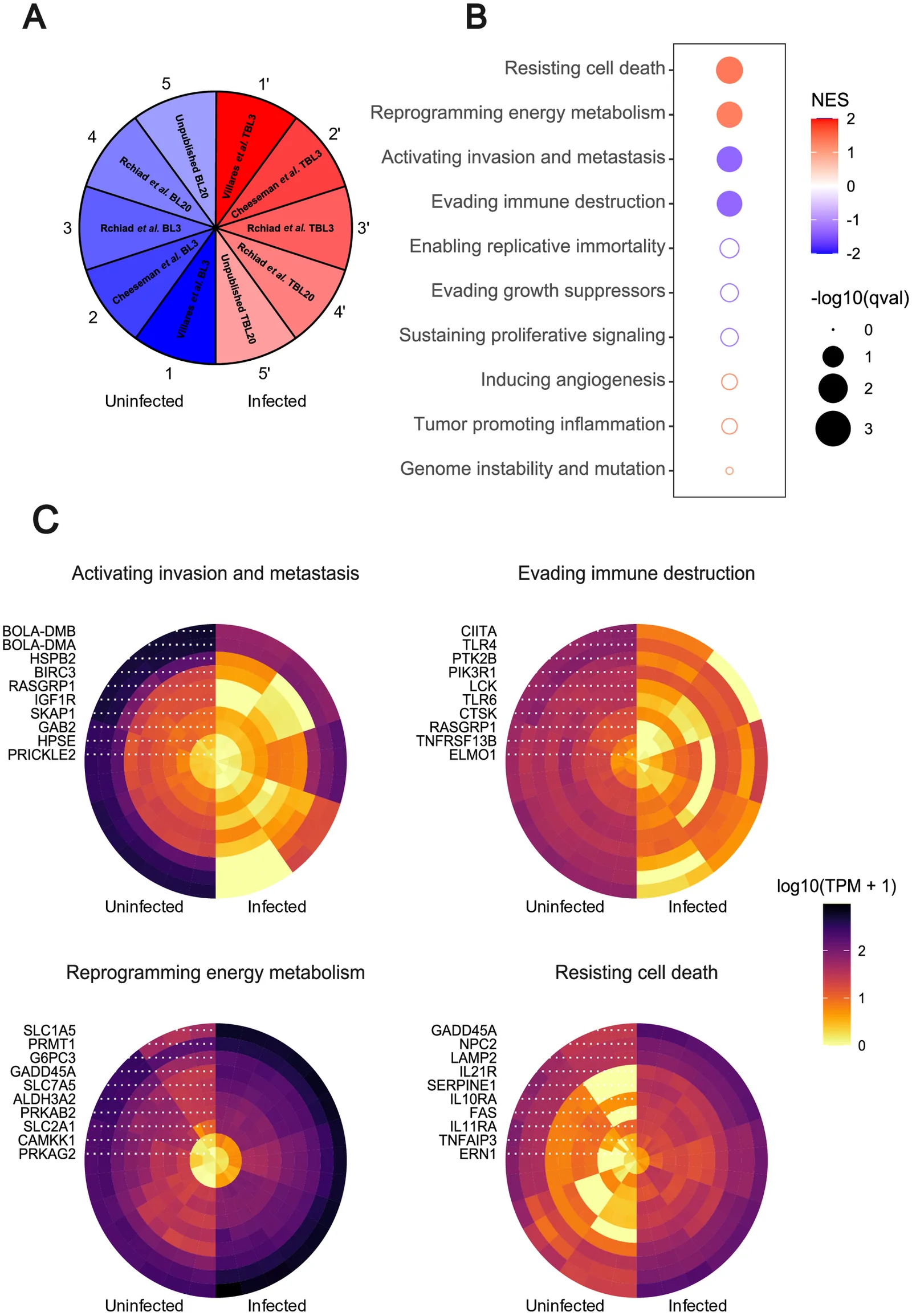
Transcriptomic analysis of *Theileria*-infected B cells highlights deregulated cancer hallmarks. (A) Schematic diagram of the published RNA-Seq datasets used for meta-analysis. A total of five datasets were used, each containing triplicate samples of uninfected (blue) cell lines or corresponding cell lines infected with *T. annulata* (red). Three datasets represent RNA-Seq analysis of uninfected BL3 cells and parasitized counterparts TBL3; Dataset 1 and Dataset 2 from our own laboratory (Villares *et al*.[38] and Cheeseman *et al*.[39], respectively) and Dataset 3 from the Langsley laboratory (Rchiad *et al*.[19]. Two datasets represent uninfected BL20 cells and parasitized counterparts TBL20; Dataset 4 from the Langsley laboratory (Rchiad *et al*. [19]) and Dataset 5 from the Rottenberg lab. We compared infected TBL3 or TBL20 cells (Datasets 1’-5’, in triplicate, on the right half of the circle shown in red) to uninfected BL3 or BL20 cells (Datasets 1–5 counterpart shown in opposite segments in blue). Polar heatmaps in all other figures use the same sample order. (B) We performed Gene set enrichment analysis (GSEA), using TPM-normalized gene counts as input, after batch correction. A custom reference database was created, using published Cancer Hallmark Genes gene sets [40]. The bubble chart represents normalized enrichment score (NES) and statistical significance, respectively. Hollow circles represent statistically non-significant results (q-val > 0.05). (C) Top 10 core enrichment genes of the statistically significant gene sets (see panel B) are shown in polar heatmaps. The left half of each heatmap represents gene expression in the 15 uninfected samples and the right half shows expression in infected samples, respectively (as displayed in panel A). The expression of each gene is shown in each concentric circle, annotated by white dots. Colour scale represents gene counts log10(transcripts per million + 1).

Infection by *T. annulata* parasites induces host genes that contribute to the transformed phenotypes [17,19]. Well-characterized examples include the matrix metalloprotease *MMP-9* gene that contributes to invasion and metastasis or the *JUN* oncogene that contributes to host cell proliferation [20,21,26,27]. These observations led to the suggestion that *Theileria* infection drives a broad transcriptional program impacting all the cancer hallmarks [13,17,18]. However, this has not been rigorously investigated and the direct contribution to different cancer hallmarks remains unclear. To comprehensively test this hypothesis, we combined transcriptome (RNA-Seq) data from 5 distinct datasets generated in three independent laboratories. This meta-dataset includes RNA-Seq data from uninfected and infected cell lines of two different B lymphocyte models. Our experimental model system (TBL3/BL3 and TBL20/BL20) allowed us to compare the parental B cells (BL3 and BL20) which are lymphoblastoid cells immortalized with bovine leukemia virus, with *Theileria*-infected paired controls (TBL3 and TBL20). Three datasets represent RNA-Seq analysis of uninfected BL3 cells and parasitized counterparts TBL3; Dataset 1 and Dataset 2 from our own laboratory (Villares *et al*. [38] and Cheeseman *et al*. [39], respectively) and Dataset 3 from the Langsley laboratory (Rchiad *et al*. [19]). And two datasets represent uninfected BL20 cells and parasitized counterparts TBL20; Dataset 4 from the Langsley laboratory (Rchiad *et al*. [19]) and Dataset 5 from the Rottenberg laboratory [Fig 1A]. PCA showed a clear divide between the infected and uninfected datasets, but we also noticed that the dataset from the Rottenberg lab is quite distinct, suggesting that there is some batch effect [S6 Fig]. This could be the result of different culture conditions, different sequencing technologies, RNA processing protocols etc. Fig 1A shows the schematic representation that we employed for all subsequent bioinformatic analysis; we compared infected TBL3 or TBL20 cells (1’-5’ datasets, in triplicate, on the right half of the circle shown in red) to uninfected BL3 or BL20 cells (1–5 counterpart datasets shown in opposite segments in blue). Combining these distinct datasets provided robust statistical analysis and reduced effects from different culture conditions or leukocytes cell lines. We initially conducted a Gene Set Enrichment Analysis (GSEA) [41] to investigate statistically significant differences between infected and uninfected B cells using the previously-established Cancer Hallmark Genes (CHG) database [40] that defined gene sets representing each of the 10 cancer hallmarks proposed by Hanahan and Weinberg in 2011 [2] [Fig 1B]. This unbiased analysis identified four hallmarks that were significantly enriched in the GSEA; we observed a positive enrichment of the hallmarks ‘*Resisting cell death*’ and ‘*Reprogramming energy metabolism’* in infected cells. This supports previous reports linked to metabolic signalling [24,25,42]. We also observed a negative enrichment of ‘*Evading immune destruction’* and ‘*Activating invasion and metastasis*’ hallmarks [Fig 1B]. There is some overlap in the genes in the different Hallmark gene sets defined by the CHG database [40]. Interestingly, we noted that several genes encoding important immunity-related proteins (e.g., *BOLA-DMB*, *BOLA-DMA*, *TLR4* and *TLR6*) were part of the core enrichment genes [Fig 1C]. Interestingly, down-regulated TLR genes were previously noted in an early microarray analysis [17]. We decided specifically to focus on the down-regulated genes in the un-enriched hallmarks, as this has not been extensively studied in the past. We explored the possibility that silenced host gene programs might contribute to an immune evasion hallmark phenotype.

### Silencing of host genes encoding innate immunity Toll-like receptors

**Fig 2:**
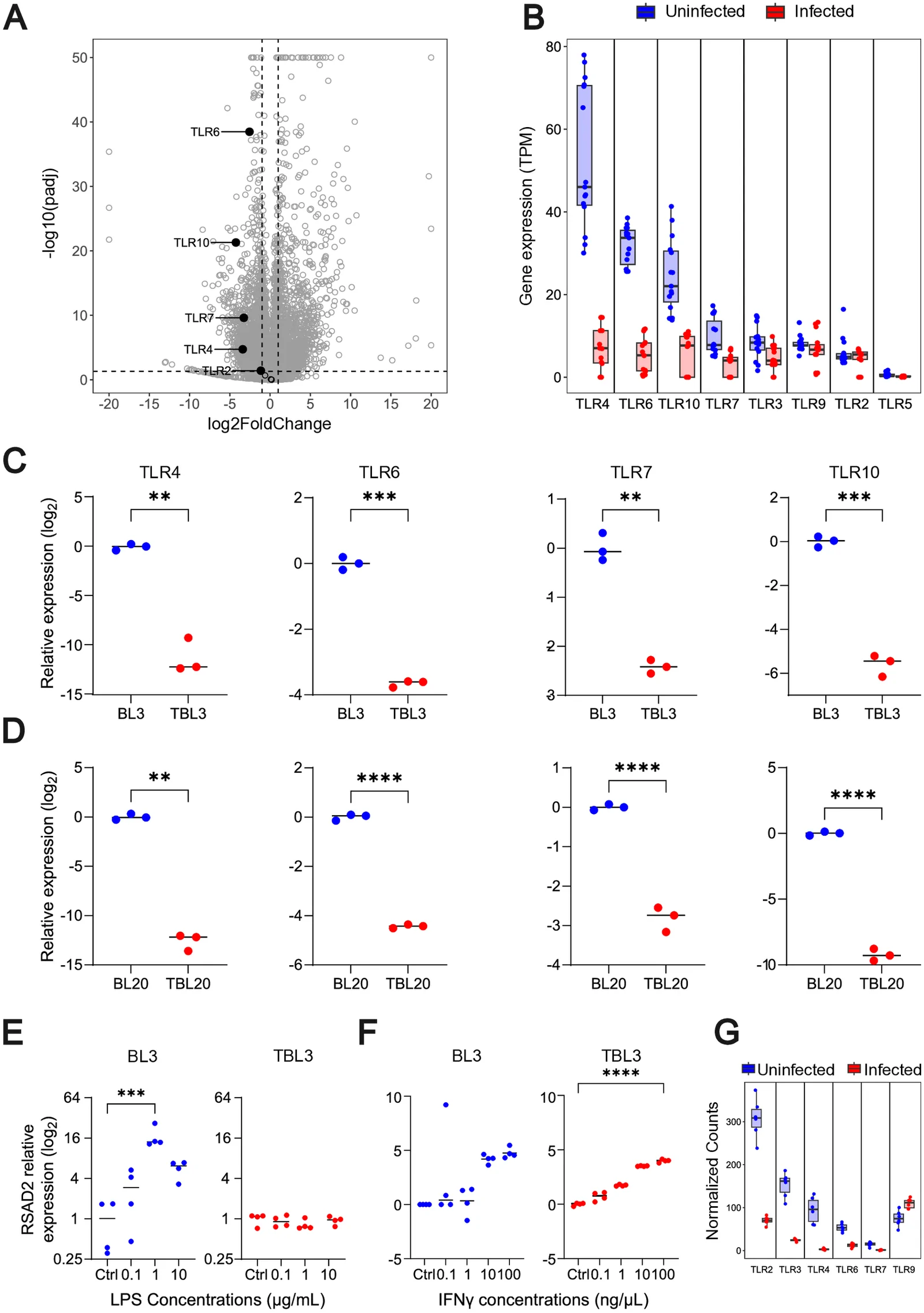
Toll-like Receptor expression and function is repressed in lymphocytes infected by *Theileria* parasites. (A) Differential expression analysis (DEA) results of combined RNA-Seq analysis, comparing all 15 infected samples with uninfected controls. Axes x,y represent log2 fold-change and -log10 adjusted p-value (Cutoffs: |log2FC| > 1, p-adj < 0.05). The down-regulated Toll-like receptors (*TLR*) are highlighted (black circles). (B) Gene expression levels (TPM-normalized gene counts) for *TLR* genes from the 15 RNA-Seq samples of the 5 datasets. Uninfected samples (BL3 and BL20) are shown in blue, and samples infected with *T. annulata* (TBL3 and TBL20) are shown in red. (C, D) Expression levels of bovine *TLR* genes (*TLR4, TLR6, TLR7* and *TLR10*) assessed by quantitative RT-qPCR PCR analysis using bovine *HPRT* gene for normalization. Expression of uninfected BL3/BL20 cells was used as reference for each gene. (C) BL3 cells (blue) compared with parasitized TBL3 cells (red). (D) BL20 (blue) cells compared with parasitized TBL20 cells (red). (E) Expression levels of bovine *RSAD2* assessed by quantitative RT-qPCR PCR analysis using bovine *H2A* gene for normalization and the BL3 expression was fixed to 1. TBL3 cells (red circles) were treated with increasing concentrations of bacterial lipopolysaccharide (LPS) for 48 hours and compared to BL3 cells (blue circles). (F) Expression levels of bovine *RSAD2* assessed by quantitative RT-qPCR PCR analysis using bovine *H2A* gene for normalization and the BL3 expression was fixed to 1. TBL3 cells (red circles) were treated with increasing concentration of IFN-γ for 48 hours and compared to BL3 cells (blue circles). (G) Comparison of *TLR* expression (normalized gene counts) in bovine T-cells after 7 days of infection with *Theileria parva* (red) to uninfected T-cells (blue) [43]. All RT-qPCR results represent three independent experiments performed in triplicate (n = 3). One-way ANOVA statistical test, Dunnett’s multiple comparison test: **p  <  0.01, ***p  <  0.001, and ****p  <  0.0001.

We noted that two TLR genes (*TLR4* and *TLR6*) were amongst the 10 most down-regulated genes in the Evading Immune Destruction hallmark dataset [Fig 1C]. Differential gene expression analysis across the collective 5 different B lymphocyte RNA-Seq datasets, showed that many *TLR* genes are down-regulated upon infection [Fig 2A]. Looking at individual transcript levels, we found that *TLR4* is the most downregulated gene in TBL3 cells infected with *T. annulata* (as previously reported [17], but several additional members of the *TLR* gene family were also downregulated [Fig 2B]. We performed quantitative reverse transcription PCR (RT-qPCR) analysis on BL3 vs TBL3 cell lines [Fig 2C] or BL20 vs TBL20 cell lines [Fig 2D] and confirmed significant down-regulation of *TLR4*, *TLR6*, *TLR7* and *TLR10* genes in parasitized cells. To demonstrate the functional consequences of TLR downregulation, we treated cells with bacterial lipopolysaccharide (LPS), the ligand of TLR4. We monitored the induction of the host *RSAD2* gene, an ISG which is also induced by TLR4 activation [44], in uninfected BL3 cells or parasitized TBL3 cells [Fig 2E]. We observed induction of *RSAD2* upon LPS treatment (0.1-10 μg/ml) in BL3 cells, but not in TBL3 cells. In contrast, treatment with interferon-gamma (IFNγ) induced *RSAD2* expression in both cell lines [Fig 2F]. These results suggest that reduced *TLR4* expression results in reduced TLR4 signalling function. The results corroborate an early observation that TBL20 cells are resistant to LPS signaling [18]. We also investigated whether infection with related *Theileria parva* parasites silences *TLR* gene expression. We queried a recent RNA-Seq dataset from *T. parva* infected T lymphocytes [43,45] and found that 7 days of infection also caused repressed *TLR* expression (specifically *TLR2*, *TLR3* and *TLR4*) [Fig 2G]. These combined observations show that parasite infection (both *T. annulata* or *T. parva*) leads to silencing of host genes encoding innate TLR receptors in B or T lymphocytes.

### Silencing of GBP inflammasome components in *Theileria*-infected lymphocytes

**Fig 3:**
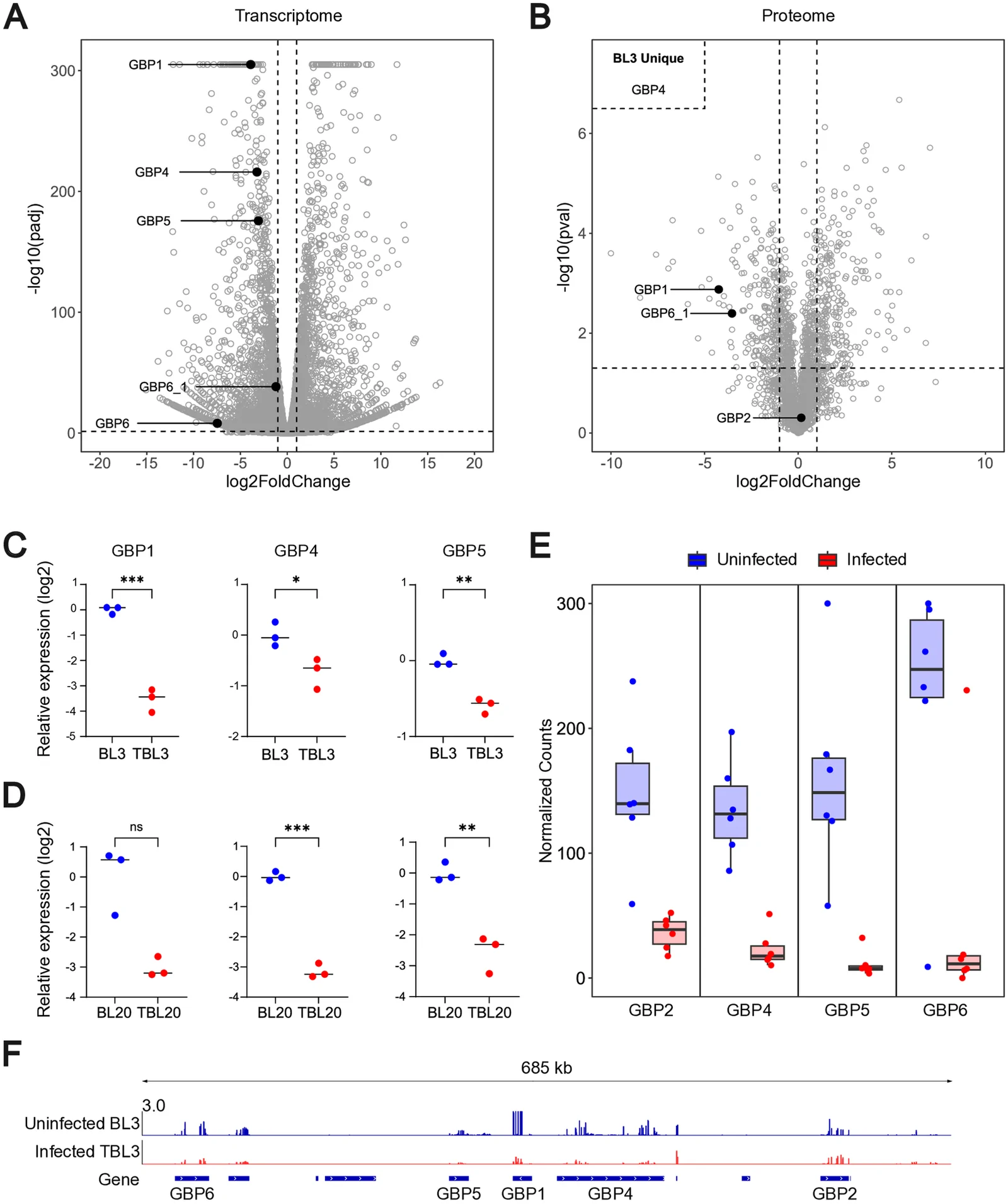
Genes encoding the GBP inflammasome components are silenced in parasitized cells. (A) Differential expression analysis (DEA) results of RNA-Seq exon analysis, comparing all 15 infected samples with uninfected controls. Axes x,y represent log2 fold change and -log10 p-value adjusted (Cutoffs: |log2FC| > 1, p-adj < 0.05). The down-regulated genes encoding guanylate binding protein (GBP) are highlighted (black circles). (B) Differential expression of proteins from whole-cell MS/MS analysis comparing triplicate samples of infected TBL3 cells with BL3 controls. Protein abundance was used as input metric to perform differential expression analysis and statistical tests. Axes x,y represent log2 fold change and -log10 p-value adjusted (Cutoffs: |log2FC| > 1, p-value < 0.05). (C, D) Expression levels of bovine *GBP* genes (*GBP1, GBP4* and *GBP5*) assessed by quantitative RT-qPCR PCR analysis using bovine *HPRT* gene for normalization and the BL3/BL20 expression was fixed to 1. (C) BL3 cells (blue circles) compared with parasitized TBL3 cells (red circles). (D) Uninfected BL20 (blue circles) cells compared with parasitized TBL20 cells (red circles). (E) Comparison of *GBP* expression (normalized gene counts) in bovine T-cells after 7 days of infection with *T. parva* (red) compare to uninfected T-cells (blue) [43]. Outlier samples with extremely high gene expression were squished to the [0,300] data range with scales::squish. (F) Screenshot of the bovine *GBP* gene locus showing RNA-Seq data for representative samples (uninfected BL3 cells in blue, infected TBL3 cells in red), using integrative genomics viewer (IGV). Base coverage was calculated with IGVtools count. All RT-qPCR results represent three independent experiments performed in triplicate (n = 3). One-way ANOVA statistical test, Dunnett’s multiple comparison test: *p  <  0.05, **p  <  0.01, ***p  <  0.001, ns = non-significant.

The guanylate binding protein (GBP) family is a group of proteins that are implicated in cell-autonomous immunity, inflammation and cancer [35]. GBPs contribute to inflammasome responses to bacterial and viral infections and are important for innate immunity. Our meta-analysis of the *T. annulata* induced transcriptome showed that parasite infection correlated with significant silencing of *GBP1*, *GBP4*, *GBP5* and *GBP6* genes [Fig 3A]. In parallel, we performed whole-cell proteomics analysis by mass spectrometry, comparing TBL3 and BL3 cells. We performed a correlation analysis to compare the whole transcriptome and proteome dataset. The correlation was R = 0.58, which is within the expected ranges for transcriptomics and proteomics. Interestingly, we observed similar down-regulation of GBP1, GBP4 and GBP6 at the protein level in whole-cell proteomics analysis [Fig 3B]. The host GBP5 protein was unfortunately undetectable in our Mass Spectrometry data. We performed RT-qPCR analysis to validate these results and we confirmed a marked down-regulation of *GBP1*, *GBP4* and *GBP5* in infected TBL3 cells compared with uninfected BL3 control cells, or in TBL20 cells compared with BL20 controls [Fig 3C-3D]. Again, we investigated whether GBP down-regulation was observed upon *T. parva* infection of bovine T lymphocytes and observed a significant reduction in *GBP* transcript levels in T cells after 7 days of infection [Fig 3E]. The bovine *GBP* genes are located in a cluster on chromosome 3 [S1 Table] and our RNA-Seq analysis showed a marked silencing of the entire gene family in TBL3 cells compared to BL3 controls [Fig 3F]. Thus, the expression of two families of innate immune genes, *TLRs* and *GBPs*, appears to be significantly silenced by *T. annulata* or *T. parva* infection in bovine lymphocytes.

### Infection leads to silencing of bovine Major Histocompatibility Complex (MHC) genes

**Fig 4:**
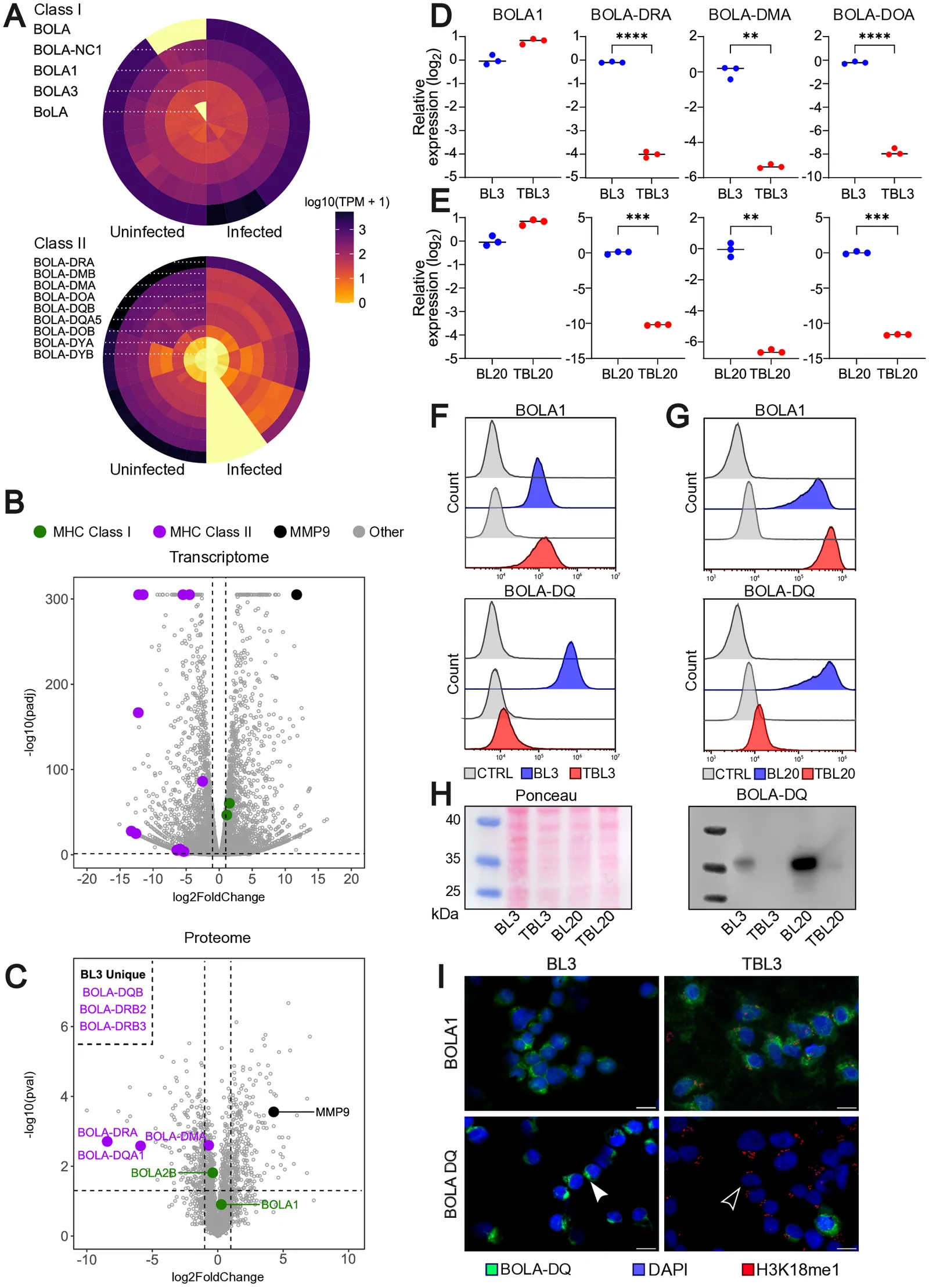
The bovine Major Histocompatibility Complex (MHC) genes are suppressed in lymphocytes infected with *Theileria* parasites. (A) Bovine major histocompatibility complex genes are shown in polar heatmaps (Class I above and Class II below). The left and right halves represent the 5 datasets uninfected (Datasets 1–5, left) and infected (Datasets 1’-5’, right) for the 15 samples (see Fig 1A). The expression of each gene is shown in each concentric circle, while the color scale represents log10(TPM + 1). (B) Differential expression analysis (DEA) results of RNA-Seq, comparing all 15 infected samples with uninfected controls. Axes x,y represent log2 fold change and -log10 p-value adjusted (Cutoffs: |log2FC| > 1, p-adj < 0.05). The down-regulated genes encoding MHC class II genes are highlighted (purple), compared to MHC Class I (green) or *MMP9* (black). (C) Differential expression of proteins from whole-cell MS/MS analysis comparing triplicate samples of infected TBL3 cells with BL3 controls. Protein abundance was used as input metric to perform differential expression analysis and statistical tests. Axes x,y represent log2 fold change and -log10 p-value adjusted (Cutoffs: |log2FC| > 1, p-adj < 0.05). Class I 5 (green), Class II (purple) and MMP9 proteins are indicated. (D, E) Expression levels of bovine *BOLA* genes (Class I *BOLA1*, and Class II *BOLA-DRA*, *BOLA-DMA* or *BOLA-DOA*) assessed by quantitative RT-qPCR PCR analysis using bovine *HPRT* gene for normalisation and the BL3/BL20 expression was fixed to 1. (D) BL3 cells (blue circles) compared with parasitized TBL3 cells (red circles). (E) BL20 (blue circles) cells compared with parasitized TBL20 cells (red circles). (F, G) Surface expression levels of bovine BOLA1 protein or BOLA-DQ assessed by flow cytometry analysis. (F) BL3 cells (blue) compared with parasitized TBL3 cells (red). (G) BL20 (blue) cells compared with parasitized TBL20 cells (red). (H) Western blot analysis of BOLA-DQ expression in infected (TBL3 or TBL20) cells compared with uninfected controls (BL3 or BL20, respectively). (I) Immunofluorescence staining for MHC I (BOLA I) or MHC II (BOLA-DQ) in uninfected cells BL3 or infected TBL3 cells. BOLA molecules are stained green (BOLA1 or BOLA-DQ), nuclei are stained with DAPI (blue) and parasite nuclei with H3K18me1 (red). Leica microscope x63 magnification; the scale bar corresponds to 10 μm. The arrow heads highlight examples of BOLA1-positive cells and BOLA-DQ-negative TBL3 cells (green labelling). Staining with the parasite-specific H3K18me1 antibody shows the intracellular parasites (red) in TBL3 cells. All RT-qPCR results represent three independent experiments performed in triplicate (n = 3). One-way ANOVA statistical test, Dunnett’s multiple comparison test: **p  <  0.01, ***p  <  0.001, and ****p  <  0.0001.

In addition to genes encoding key components of the innate immune response, our cancer hallmarks analysis identified genes encoding the bovine Major Histocompatibility Complex (MHC) as deregulated upon infection [Fig 1C]. There was no significant change in the expression of genes encoding MHC Class I (referred to as BoLA or BOLA) cell surface proteins, but several MHC Class II *BOLA* genes were significantly suppressed across the five B lymphocyte datasets [Fig 4A, lower panel]. We performed transcriptomic analysis of RNA-Seq datasets [Fig 4B] and parallel whole-cell proteomic analysis of TBL3 and BL3 cells [Fig 4C]. Both the transcriptomics and proteomics analyses independently showed a down-regulation of MHC-BOLA Class II proteins [Fig 4A-4C]. We performed RT-qPCR analysis in TBL3/BL3 cells [Fig 4D] or TBL20/BL20 cells [Fig 4E] and confirmed that infection led to silencing of *BOLA-DRA* expression, *BOLA-DMA* and *BOLA-DOA* genes, compared to uninfected BL3 or BL20 cells, but did not affect the *BOLA1* Class I gene [Fig 4D-4E]. We then performed flow cytometry analysis and also confirmed down-regulation of the Class II BOLA (BOLA-DQ/DR) proteins on the cell surface in TBL3 cells [Fig 4F] or TBL20 cells [Fig 4G], but no changes in the BOLA1 MHC Class I. We performed western blot analysis of extracts from TBL3 or TBL20 cells and confirmed down-regulation of MHC II proteins in parasitized cells [Fig 4H]. Finally, immunofluorescence analysis detected reduced MHC Class II surface expression in TBL3 cells compared to BL3 cells [Fig 4I]. In conclusion, these experiments show that there is a significant and selective silencing of the expression of BOLA-MHC Class II molecules, at both the transcript and protein levels, in parasitized lymphocyte cells.

### Immune gene expression in other *Theileria* infection models *in vitro* and *in vivo*

**Fig 5:**
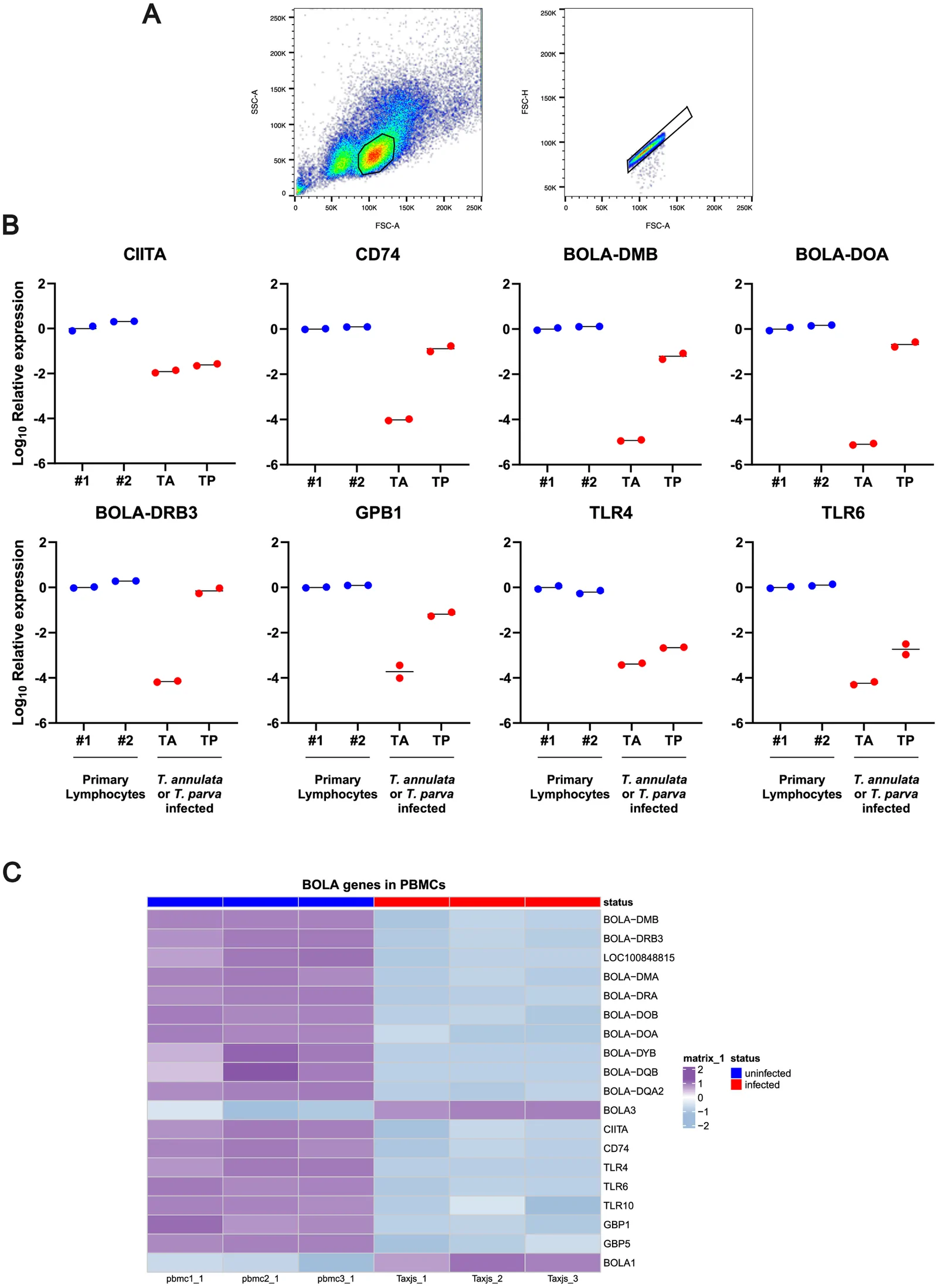
Differential expression of innate immune genes in primary bovine lymphocytes and *Theileria*-infected cells. (A) Representative flow cytometry gating strategy used to isolate lymphocytes from cryopreserved bovine PBMCs and *Theileria*-infected blood-derived isolates. Cells were gated based on FSC/SSC characteristics followed by singlet discrimination using FSC-A versus FSC-H. (B) RT-qPCR analysis of immune signaling in primary bovine lymphocytes and *Theileria*-infected isolates. Expression values are shown as log₁₀ relative expression to PBMCs normalized to H2A. Each point represents a biological replicate. (C) Heatmap showing the expression of selected *BOLA* and immune-related genes in uninfected bovine PBMCs and *Theileria annulata*-infected leukocytes. RNA-Seq data were obtained from the publicly available dataset generated by Yijun Chai *et al*. [46] and reprocessed using the transcriptomic analysis pipeline described in this study. Expression values are displayed as row-scaled normalized counts (z-scores), with higher and lower expression indicated by red and blue, respectively.

Our experimental model system (TBL3/BL3 and TBL20/BL20) is rather particular, as the parental B cells (BL3 and BL20) are lymphoblastoid cells which are infected with bovine leukemia virus. We favoured this system for our multi-omics analysis because the parental lines serve as strong paired controls. However, in order to overcome the potential caveats in this system we investigated the regulation of immune genes in additional naturally infected cell lines comparing them with PBMC isolated from uninfected cows. We selected B lymphocytes and compared these with two independent infected cell isolates from infected cattle [Fig 5A]. We observed that infection (*T. annulata* or *T. parva*) correlated with the silencing of *TLR*, *GBP* and *BOLA* genes in these cells compared with the PBMC baseline levels [Fig 5B). These data support the conclusion that immune gene silencing is not an artefact of the TBL3 or TBL20 cell models.

To explore whether the changes we observed in these different cellular models *in vitro* are reflected during normal parasite infection *in vivo*, we sought data from parasite infections in cows. Our analysis of a dataset from *T. parva* infected T lymphocytes after 7 days of cattle infection [43,45] showed that *TLR* and *GBP* genes were significantly repressed *in vivo* [Figs 2G and 3E]. The silencing of *BOLA-MHC* genes was less clear, but this could reflect differences between T and B lymphocyte target populations. A recent study reported transcriptomic profiling during *T. annulata* infections in cattle [46]. We re-analysed these data focusing on the relative expression of *TLR*, *GBP* and *BOLA* genes. We observed a marked reduction in the expression of all these immune genes, but no changes in the expression of control *BOLA1* genes [Fig 5C]. These data from two independent datasets obtained from *Theileria*-infected cows provide strong support for the silencing of immune genes during *in vivo* infections.

### The epigenomic landscape of the promoters of immune genes

**Fig 6:**
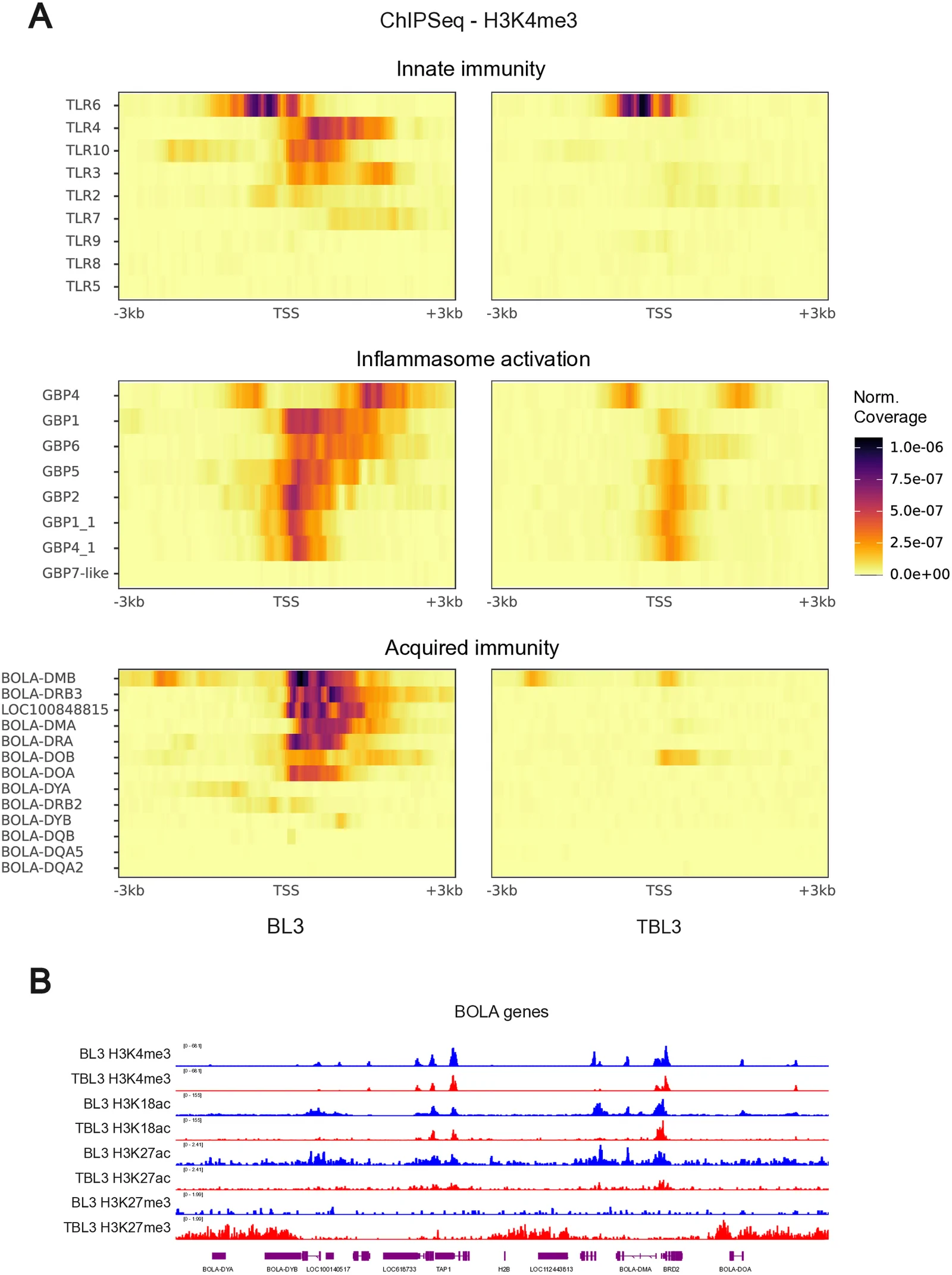
Loss of activating epigenetic modifications on the promoters of immune genes. (A) The epigenetic landscape, as assessed by the ChIP-Seq [39] signal intensity of H3K4me3 histone modification, over the promoters of immune genes in infected TBL3 cells (right panels) or uninfected BL3 cells (left panels). Axis x represents the transcription start site of each gene (TSS), and 3kb flanks upstream and downstream. Signal intensity was summarized in 10 bp bins (600 bins total) with Deeptools and averaged over two samples after normalization. (B) Representative screenshot showing one of the MHC class II loci, in integrative genomics viewer (IGV), highlighting the enrichment of H3K4me3, H3K18ac, H3K27ac or H3K27me3 modifications in infected TBL3 cells (red) or uninfected BL3 controls (blue). Base coverage was calculated with IGVtools count.

**Fig 7:**
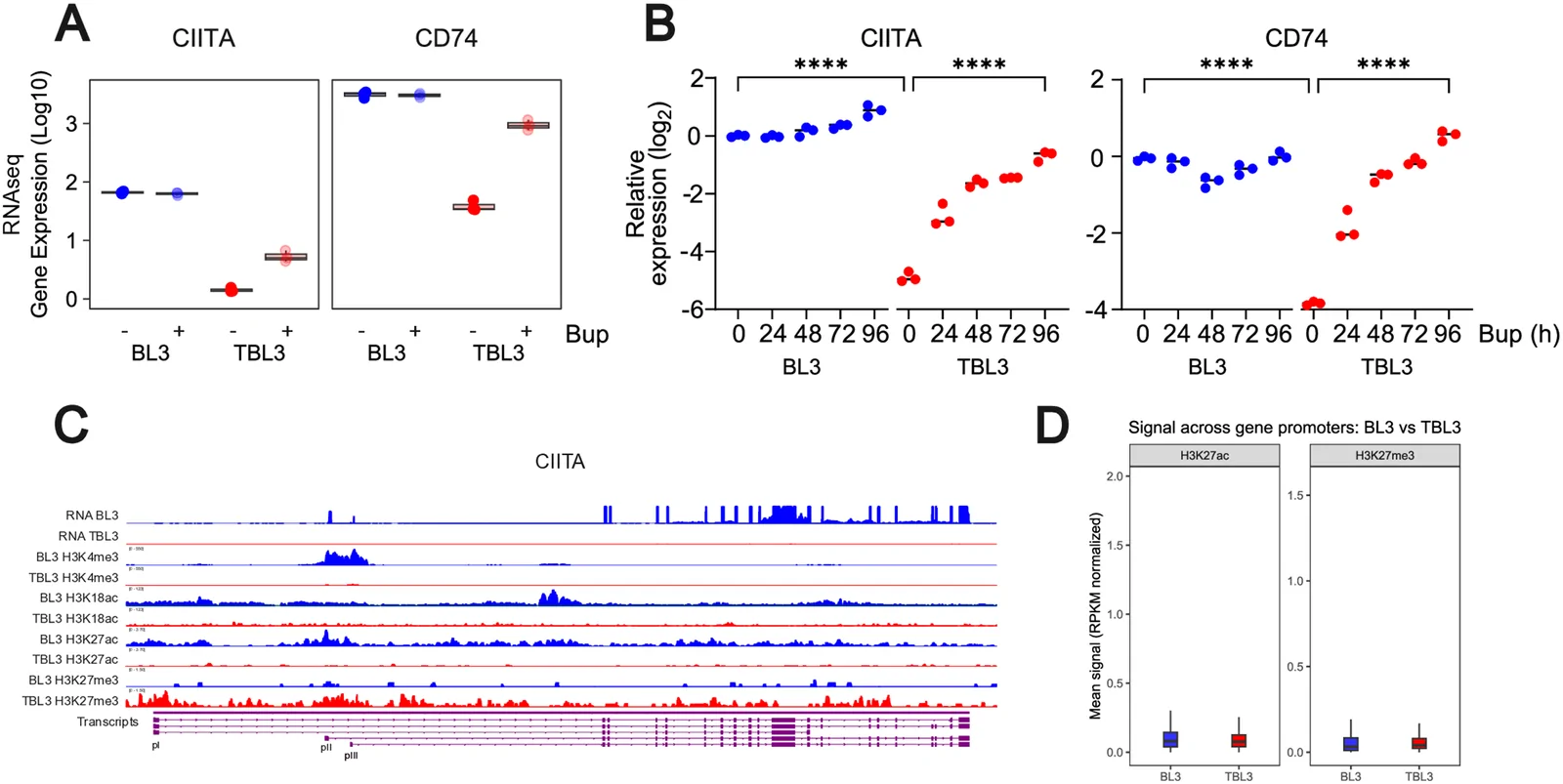
CIITA, the master regulator of MHC class II genes, is silenced in *Theileria*-infected lymphocytes. (A) Expression of the bovine *CIITA* gene (TPM-normalized gene counts) and its target gene, *CD74*, in infected (TBL3) cells (red) compared with uninfected control (BL3) cells (blue). Cells were treated with the anti-parasite drug Buparvaquone [39] (Bup) for 24 hours. (B) Time-course experiment following the rescue of *CIITA* and *CD74* expression in TBL3 cells (red circles) following treatments with the Buparvaquone (Bup) drug for up to 96 hours, compared with uninfected cells (blue circles). (C) Representation of the bovine *CIITA* locus highlighting RNA-Seq data [39] for uninfected BL3 cells (blue) or infected TBL3 cells (red) and corresponding H3K4me3, H3K18ac, H3K27ac or H3K27me3 chromatin immunoprecipitation ChIP-Seq or Cut’&’Run analysis in integrative genomics viewer (IGV). Base coverage was calculated with IGVtools count. The four *CIITA* promoters (pI to pIV) are indicated. (D) Graph showing the global signal of H3K27ac or H3K27me3 across all gene promoters in BL3 or infected TBL3 cells. All RT-qPCR results represent three independent experiments performed in triplicate (n = 3). One-way ANOVA statistical test, Dunnett’s multiple comparison test: ****p  <  0.0001.

Epigenetic changes can contribute significantly to transcriptional profiles of cancer hallmarks [28]. We previously generated chromatin immunoprecipitation data of epigenetic modifications in parasitized TBL3 cells and uninfected BL3 controls [39]. Now, we investigated whether the reduced expression of the host immunity genes was associated with changes in the epigenetic features of their promoters. We observed that the enrichment of trimethylated histone H3K4 (H3K4me3) was reduced around the promoters of many of the repressed immunity genes, including genes encoding TLR, GBP and BOLA Class II proteins [Fig 6A]. We also observed decreases in the promoter enrichment of H3K18 acetylation (H3K18ac), another activating epigenetic modification [Fig 6B]. This was specific to the promoters of the immunity genes; for example, adjacent genes did not show changes in the epigenetic marks on their promoters [Fig 6B]. To further investigate the epigenetic reprogramming upon parasite infection, we performed a new Cut’&’Run analysis using antibodies recognizing the activating H3K27ac modification or the repressive H3K27me3 mark. This analysis showed that the levels of H3K27ac decrease on silenced *BOLA* genes in TBL3 cells [Fig 6B]. Interestingly, the loss of activating marks on the silenced genes was associated with an increase in the H3K27me3 repressive modification [Fig 6B]. The enrichment of H3K27me3 specifically on *BOLA* gene promoters does not reflect a general genome-wide repression [Fig 7]. Thus, *T. annulata* parasites appear to remodel the epigenetic features of the promoters of host genes encoding innate and adaptive immunity effectors, involving a switch from activating histone modifications to repressive modifications.

### Silencing of the MHC Class II transactivator CIITA gene

The genes encoding human MHC Class II proteins [31] are regulated at the transcriptional level by the MHC Class II transactivator CIITA (also known as NLRA) [47,48]. We investigated the expression of the bovine *CIITA* gene and observed that it was significantly down-regulated in our RNA-Seq analysis of infected TBL3 cells compared to BL3 cells [Fig 7A]. Furthermore, we also observed down-regulation of the *CD74* gene, a characterized CIITA target gene that encodes the invariant chain of MHC Class II [Fig 7A]. The theilericidal drug Buparvaquone can eliminate intracellular parasites and reverse some aspects of the transformed phenotype in host cells [17,22]. Analysis of our RNA-Seq dataset showed that treatment with Buparvaquone (50 ng/ml for 24 hours) could partially rescue expression of the *CIITA* and *CD74* genes in TBL3 cells [Fig 7A]. To explore the extent of the Buparvaquone rescue, we treated cells for longer time-points and followed the expression of *CIITA* and *CD74* transcripts. We observed that drug treatment for up to 96 hours led to a gradual rescue of the of *CIITA* and *CD74* levels in TBL3 cells [Fig 7B]. An earlier microarray study had shown that some deregulated genes are rescued following Buparvaquone treatment, while others are not [17]. The *CIITA* gene is known to use several alternative promoters [48]. Chromatin analysis in BL3 cells suggested that the pII and pIII promoters (known to be a B cell promoter) are the major transcriptional start sites in these cells and are covered by a methylated H3K4me3 enriched mark, as well as acetylated H3K27ac (and to a lesser extent H3K18ac) [Fig 7C]. In TBL3 cells these activating epigenetic marks are lost on the pII and pIII promoters, correlating with silencing of *CIITA* transcripts [Fig 7C]. This loss is accompanied by a switch to H3K27me3 repressive marks, supporting the notion that the parasite actively silences this gene [Fig 7C]. This enrichment for the H3K27me3 modification is not the result of a global increase in H3K27me3 levels across the bovine genome upon infection [Fig 7D]. These combined results suggest that the reprogramming of epigenetic modifications on the promoter of the MHC Class II transactivator *CIITA*, leads to reduced expression of *CIITA* and its target gene *CD74* in parasitized cells and that these effects could be reversed by treatment with the Buparvaquone drug. We also investigated the level of expression of *CIITA* and *CD74* genes in a dataset from cows infected with *T. annulata*. Here again, the loss of *BOLA* gene expression correlated with silencing of *CIITA* and *CD74* genes [Fig 5C], supporting the suggestion that CIITA controls *BOLA* gene expression upon infection in cattle.

### Rescue of the expression of immunity genes by theilericidal drugs

**Fig 8:**
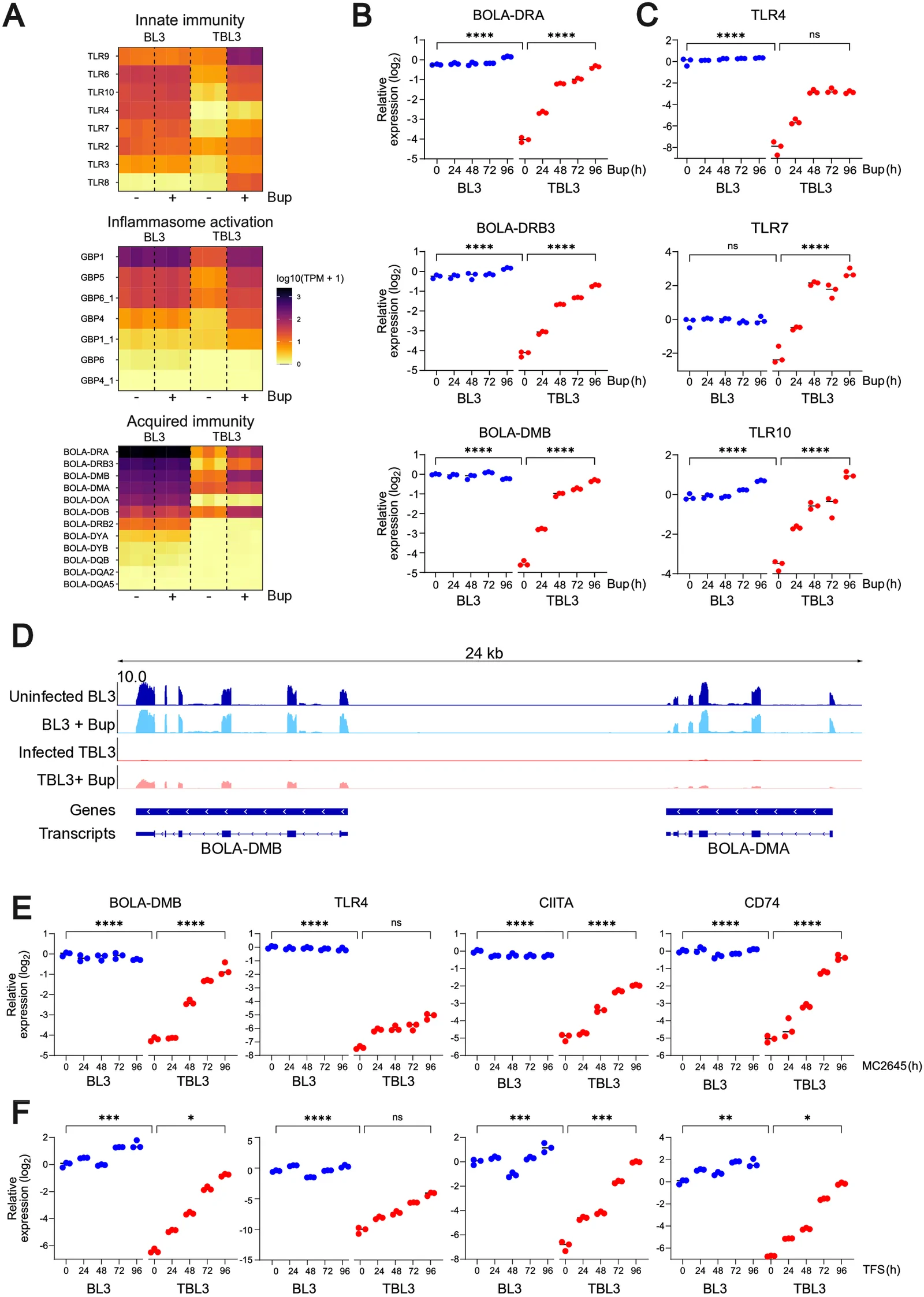
Theilericidal drugs rescue the expression of innate and adaptive immune genes. (A) Expression analysis presented as heatmaps showing TPM-normalized exon counts for immune genes. The Fig represents the Cheeseman *et al.* 2021 [39] bulk RNA-Seq datasets following treatment for 24 hours with Buparvaquone (Bup). Genes with no assigned reads were omitted. Color scale represents log10(TPM + 1). (B-C) Time-course RT-qPCR experiments showing the rescue of the expression of MHC Class II *BOLA* genes (B) or *TLR* genes (C) in TBL3 cells (red circles) following treatments with Buparvaquone (Bup) for up to 96 hours (and compared to BL3 cells, blue circles). (D) Representative screenshot showing two genes in the MHC Class II BOLA locus highlighting RNA-Seq data of uninfected BL3 cells (blue) or infected TBL3 cells (red) and partial rescue at 24 hours following treatment with Buparvaquone (Bup), in integrative genomics viewer (IGV). Base coverage was calculated with IGVtools count. (E-F) Time-course RT-qPCR experiments showing the rescue of the expression of MHC Class II *BOLA-DMB, TLR4, CIITA* and *CD74* genes in TBL3 cells (red circles) compared to BL3 cells (blue circles) following treatments with two anti-*Theileria* drugs; compound MC2645 (panel E) or Trifloxystrobin (TFS, panel F) for up to 96 hours. All RT-qPCR results represent three independent experiments performed in triplicate (n = 3). One-way ANOVA statistical test, Dunnett’s multiple comparison test: **p  <  0.01, ***p  <  0.001, and ****p  <  0.0001, ns = not statistically significant.

To investigate the reversibility of the silenced immunity genes and to explore the role of changes in *CIITA* expression, we tested treatment with the theilericidal drug, Buparvaquone. Analysis of our bulk RNA-Seq data showed that the silencing of *TLR*, *GBP* and *BOLA* genes in TBL3 cells could be partially reversed by treatment with Buparvaquone for 24 hours [Fig 8A]. We confirmed these results using RT-qPCR analysis over a longer time-course. Treatment with Buparvaquone lead to a gradual rescue of expression of *BOLA* Class II genes and *TLR* genes in TBL3 cells [Fig 8B]. These results correlate well with the effect of the drug on rescue of the expression of the CIITA MHC activator [Fig 7]. This reversibility can also be seen clearly in a screenshot of the BOLA Class II locus [Fig 8D]. It has been well characterized that Buparvaquone treatment leads to apoptosis of host cells as well as parasite removal [17,26]. We therefore performed treatment with two other drugs that eliminate intracellular parasites through different modes of action. The Trifloxystrobin [16] compound targets parasite CytB and also cause host cell death, whereas the newly identified MC2645 [38] removed parasites by autophagy without impacting host cell survival. Treatment with both these drugs also rescued the expression of *CIITA* and *BOLA* Class II, *TLR4* and *CD74* expression in TBL3 cells [Fig 8E and 8F]. These results suggest that the silencing of host immune genes by the parasite is not correlated with apoptosis, but is linked to the presence of the parasite. Silencing can be mainly accounted for by the down-regulation of epigenetic modifications on the immunity gene promoters and the modulation of the transcription factor CIITA.

## Discussion

Transformation of bovine leukocytes by intracellular *T. annulata* parasites is associated with extensive changes in gene expression that contribute to cancer phenotypes [13,17,19]. Here, we described an integrated multi-omics approach to conduct an unbiased investigation of the impact on Cancer Hallmarks [1,2,40]. This led us to discover a general silencing of both innate and adaptive immune response genes in bovine cell lines infected with *T. annulata* and *T. parva*. Our analysis of the host transcriptome, proteome and epigenome showed that genes associated with the “Evading immune destruction” cancer hallmark are reversibly silenced; these include innate immunity TLRs, components of the inflammasome and the adaptive immune molecules of the bovine MHC/BOLA Class II gene cluster. This contributes to our appreciation of the epigenetic contribution to the complexity underlying cancer hallmark regulation [28] and underlies the potential targeting of epigenetic enzymes to treat parasitic infections [9].

Our analysis of bulk RNA-Seq transcriptomes was enhanced by comparing five available datasets of TBL3 or TBL20 parasitized cells compared with uninfected controls (BL3 or BL20, respectively). Unbiased gene set enrichment analysis (GSEA, see Fig 1) of the five datasets highlighted the downregulation of selected immunity genes, consistent with immune dampening upon infection. The Toll-like receptors are critical sensors and effectors of the innate immune response and are crucial for engaging intracellular pathogens [33]. Four of these receptors (TLR4, TLR6, TLR7 and TLR10) were down-regulated in lymphocytes infected with *T. annulata* or *T. parva*. This extends the previously observed suppression of *TLR4* and LPS responses in *T.* annulata-infected lymphocytes revealed by microarray analysis [17,18]. Little is known about the transcriptional regulation of the *TLR* gene family and the genes are located at different genomic loci (see S1 Table). It will be interesting to investigate how the parasite broadly affects the expression of TLR family genes. In contrast, the genes encoding the family of GBP innate immunity sensors are clustered together on chromosome 3. Many pathogens, particularly viruses, target GBP proteins to block inflammasome innate immune defenses, but there are few examples of transcriptional suppression of *GBP* genes by invading non-viral pathogens [35]. These differences in innate immune gene expression from our transcriptome analysis were confirmed by RT-qPCR analysis in TBL3 and TBL20 cell lines, and in whole-cell proteomic analysis. Although we cannot provide a clear mechanistic insight into how these two gene families are downregulated, the reversibility by several theilericidal drugs (Buparvaquone, Trifloxystrobin, and compound MC2645) and the observed changes in the epigenomic features around their promoters, supports the suggestions that epigenetic changes could account for the impacts on host gene expression [9]. As the MC2645 compound removes parasites by autophagy without host cell apoptosis, we conclude that the reversibility is strictly linked to the presence of the parasite, rather that cell death.

The major histocompatibility complex is encoded by the BOLA (Bovine leukocyte antigen) gene clusters in the cow genome (S1 Table). Comparison of RNA-Seq datasets in the two *T. annulata*-infected cell lines showed a robust suppression of the genes encoding MHC class II, but no effect on MHC Class I genes. These transcriptomic observations were confirmed by whole-cell proteomics analysis of TBL3 cells and analysis of cell-surface expression by flow cytometry or immunofluorescence. Our analysis of epigenome features again suggested that the *BOLA II* genes are subject to epigenetic silencing upon *T. annulata* infection. We also noted a marked downregulation of the host *CIITA* gene which encodes the master regulator of MHC class II expression [48]. Downregulation of CIITA in infected cells correlated with a depletion of activating epigenetic marks (namely H3K27ac, H3K4me3 and to a less extent H3K18ac) over its active promoter regions (pII and pIII). This was accompanied by an increase in repressive H3K27me3 modification, suggesting that the epigenetic switch regulates immune gene silencing. As CIITA is required for the expression of MHC/BOLA Class II genes, we favor a model in which the down-regulation of CIITA protein and the changes in the chromatin structure, lead to parasite repression of the adaptive immune response genes. The regulation of CIITA and MHC Class II genes by H3K27 methylation via the EZH2 methyltransferase of the Polycomb repressor complex PRC2 has been observed in human cancer cells [49]. We show that treatment with drugs (Buparvaquone, Trifloxystrobin, or compound MC2645) that reduce parasite load, led to a rescue of BOLA gene expression (independently of apoptosis). It is possible that this reversibility is explained by the CIITA regulator which could act as a scaffold to recruit chromatin modifying enzymes and rescue *BOLA II* gene expression. Previous transcriptomic studies also described sets of genes that were reversed by Buparvaquone and genes whose expression was not rescued by drug treatment [29]. The mechanisms that explain why some loci are reversible and others are not merits further investigation.

Our study focused on the use of BL3/TBL3 and BL20/TBL20 cell models as pairs of infected and uninfected control cells. This is a powerful system to investigate the impact of *T. annulata* parasites on host gene expression. These results were strengthened by the reversible silencing using three different drug treatments that eliminate intracellular parasites through different modes of action. However, it was important to validate our finding with cells from naturally infected cows. We confirmed the down-regulation of the host immune genes comparing additional naturally infected isolates with PBMC controls. We also investigated two transcriptome datasets from infected cattle. In both cases, infection with *T. parva* or *T. annulata* led to immune gene silencing after one week of infection. These results support our conclusions for a general wide-spread silencing of immune gene families by transforming *Theileria* species. Further experiments will be necessary to determine whether the down-regulation seen in cows is directly observed in infected B cells, for example by single-cell RNA sequencing. At this point, we can only speculate about how downregulating MHC Class II could disrupt the CD4-mediated licensing of the immune response, effectively blunting helper function to allow CD8 + T-cells to function efficiently. In this way parasites may modulate the quality of the T-cell response to prevent a robust, sustained adaptive response. This combines with a defective innate immune reponse (TLR and inflammasome) to permit effective parasite persistence. The preservation of BOLA Class I expression suggests that rather than a generalized immune evasion, the repression of immuno-modulatory genes could remodel the functional identity of infected leukocytes, potentially altering antigen-presenting capacity and inflammatory signalling while maintaining susceptibility to CD8 ⁺ T-cell recognition. It is also important to note, that the effects of *Theileria* infection can be highly variable and complex. It will be interesting to explore the dynamics of immune gene modulation in other infection models *in vitro* and *in vivo*.

The coordinated down-regulation of three families of innate response genes by *T. annulata* (and *T. parva*) infections is quite remarkable. Viral and bacterial infections often target components of the innate and adaptive immune system [33,34,50,51]. This can involve selective degradation of specific protein components. To our knowledge, this is the first example of wide-scale epigenetic silencing of genes encoding TLRs, GBPs and BOLA Class II upon parasite infection. The generalized transcriptional silencing of these gene families implies a broad epigenetic rewiring by *Theileria* parasites to mount an effective evasion of the host immune responses. Further studies will determine the mechanisms for this immune gene repression and the relative contribution of chromatin modifying enzymes and/or transcriptional regulators (such as CIITA).

## Materials and methods

### Read mapping & feature counting

Raw RNA-Seq & ChIP-Seq fastq files were mapped to the fused *Bos taurus* (ARS-UCD1.2) and *Theileria annulata* (GCF_000003225.3) genomes after concatenating the respective genome files in order to discriminate host and parasite reads. Read mapping was performed with HISAT2, as part of the BiBs modified RASFlow [52] pipeline (github: parisepigenetics/RASflow_EDC). For RNA-Seq alignment files, feature counting was performed to identify mapped reads overlapping genomic features. For this part of the pipeline, featureCounts was used to extract gene counts for each feature, using a likewise concatenated genome annotation file. For analyses pertaining to the GBP gene locus (Figs 2 and 8), exon counts were used instead to account for overlapping features.

### Gene set enrichment analysis

Gene counts of all RNA-Seq datasets [19,38,39] were TPM-normalized and batch-corrected with limma v3.50.0 [53]. These were used as input for GSEA v4.3.2 [41,54] to compare infected to uninfected samples. Enrichment was calculated in reference to a custom GSEA gene set database, according to the cancer hallmarks gene sets suggested by Zhang *et al*.[40]

### Differential expression analysis

Differential expression analysis (DEA) was performed with DESeq2 v1.52.0 [55]. Unnormalized gene counts of all datasets were used as input to compare the expression of infected to uninfected samples, as DESeq2 performs internal normalization. We used DESeq2 (design= ~ batch+ctrl+condition) to model for batch effect and different control cell lines (BL3 and BL20).

### Epigenomic analysis

Reads aligned over the promoters of genes were counted in 10 base pair bins (600 bins total, 3kb flanks) using Deeptools v3.5.6 [56]. The per-bin signal intensity was normalized to total promoter signal and subsequently averaged between the 2 samples per condition. The bigwig tracks were generated using deeptools’ bamCoverage with the parameters as ‘—normalize Using RPKM --binSize 10 --smoothLength 40.’ The ChIP-Seq data for H3K4me3 and H3K18ac were previously reported [39].

### Data visualization

Processed RNA-Seq, ChIP-Seq and proteomics data were visualized in R v4.1.1 using ggplot2 v3.4.4 [57]. To visualize genomic tracks, base coverage for each alignment was calculated with IGVtools v2.5.3 count. The produced  .tdf files (window size of 7 bp) were visualized on IGV v2.13.2 [58] using  .tdf normalization. Quantitative reverse transcription PCR (RT-qPCR) results were visualized with Graphpad Prism v10.2.2. Final arrangement of figures was done with InkScape v0.92.1.

### Cell culture

All bovine cell lines and growth conditions were previously documented [19,39]. TBL3 and TBL20 cells were derived from *in vitro* infection of the spontaneous bovine B lymphosarcoma cell lines, BL3 and BL20, with Hissar stock of *T. annulata*. Cells were cultured in RPMI 1640 (Gibco-BRL), supplemented with 10% heat-inactivated fetal bovine serum (FBS), 4 mM L-glutamine, 25mM HEPES, 10mM β-mercaptoethanol and 100mg/ml of penicillin/streptomycin in a humidified 5% CO_2_ atmosphere at 37°C. Cell lines were kindly provided by G. Langsley (Université Paris Cité, France) and K. Woods (University of Bern, Switzerland).

### Processing of Bovine PBMCs and *Theileria*-Infected Cell Isolates

Cryopreserved bovine peripheral blood mononuclear cells (PBMCs) obtained from healthy cattle and cryopreserved cell isolates recovered from the peripheral blood of *Theileria annulata*-infected cattle were thawed rapidly and transferred into pre-warmed culture medium supplemented with 40% fetal bovine serum (FBS). Cells were pelleted by centrifugation to remove cryopreservation medium and washed with phosphate-buffered saline (PBS). To reduce cell clumping resulting from extracellular DNA released during thawing, cell suspensions were treated with DNase I before further processing.

Following DNase I treatment, cells were resuspended in sorting buffer and lymphocytes were identified and purified by fluorescence-activated cell sorting (FACS) based on their forward scatter (FSC) and side scatter (SSC) characteristics. Sorted lymphocyte populations were collected for downstream molecular analyses. Total RNA was extracted from sorted cells and reverse transcription quantitative PCR (RT-qPCR) was performed as described in the corresponding sections below.

### Drug treatments

Bovine cell lines were treated with drugs under the following conditions (as previously described): Buparvaquone was used at 50 ng/ml for 24 or 48 h; compound MC2645 and Trifloxystrobin were used at 4 μM [16,38]. Medium and drugs were refreshed every 24 hours for time-course experiments and cells were collected for each time-point.

### Gene expression analysis (RT-qPCR)

Total RNA was extracted from cell cultures using a Nucleospin RNA extraction kit (MachereyNagel) following the manufacturer’s protocol. 5 μg of total RNA were reverse transcribed (RT) with Superscript IV Reverse transcriptase Kit (Invitrogen). Real-time quantitative RT-qPCR was performed to analyze relative gene expression levels using PowerTrack SYBR Green Master Mix (Applied Biosystems) following the manufacturer’s protocol. Relative expression values were normalized with bovine housekeeping genes *H2A*, *HPRT1* or *HSP70*. The primer sequences used are listed in S2 Table.

### Flow cytometry analysis

Cells were harvested, washed in PBS and fixed for 10 min at RT using 4% paraformaldehyde. Samples were washed in PBS again and subsequently permeabilized (permeabilization buffer: 5% FBS, 0.02% sodium azide, 0.1% saponin and 20% heat-inactivated horse serum in PBS) for 30 min at RT. After permeabilization, samples were incubated for 30 min at 4°C with primary antibodies against BOLA1 (MHCI IL-A88, 1:4000 dilution in permeabilization buffer [59] and against BOLA-DQ (MHCII-DQ clone CC158, 1:2000 dilution in permeabilization buffer [60]. Cells were washed twice with PBS prior to incubation with a secondary antibody for 30 min at 4°C (Alexa Fluor 568, Goat anti-Rabbit IgG (H + L)). Stained cells were washed twice more in permeabilization buffer followed by one wash in PBS and a further fixation step with 1% PFA before analysis by flow cytometry. Antibodies recognizing bovine BOLA proteins were a kind gift from Timothy Connelley (Roslin Institute, Scotland). S1-S5 Figs show the gating strategy, the dot plots, the contour plots, the controls used for each experiment in BL3/B20 and TBL3/TBL20. The cytometer used is CytoFLEX SRT and analysis were done with Kaluza Analysis 2.2 software.

### Western blot analysis

Cells were collected and proteins were extracted with Laemli lysis buffer, then resolved by running gels SDS-PAGE 4–12%, and transferred to nitrocellulose membranes, before being incubated overnight at 4°C with the primary antibody against BOLA-DQ (MHCII-DQ clone CC158, 1:2000 dilution) followed by secondary antibodies produced against mouse antibodies. The membrane was blocked in milk 5% PBSt for 45min and antibodies were diluted in milk 1% PBSt.

### Immunofluorescence and microscopy

B cells (BL3 & TBL3) were washed with PBS and 100,000 cells per slide were centrifuged with Cytospin (10min at 1000 × g) to stick to the slide. Cells were fixed in 3.7% paraformaldehyde for 10min then permeabilized in 0.2% Triton-PBS for 10min. Cells were blocked with PBS 0.2% Tween (PBST)−1% BSA for30min. The primary antibodies targeting respectively BOLA1 and BOLA-DQ were diluted in blocking solution and incubated for 45min at the following dilutions: MHCI ILA88 1:2000 and MHCII-DQ CC158 1:2000. Cells were washed three times with PBST and incubated with secondary antibody for 30min at 1:1000 dilution (Alexa Fluor 488 goat anti-mouse IgG (H + L)). Cells were washed three times with PBST and finally mounted on glass coverslips, adding mounting medium containing DAPI. Samples were analyzed using a Leica DMI 6000 epifluorescence microscope. Images were generated and processed using Metamorph and ImageJ software.

### Whole-cell proteome (WCP) sample preparation and mass spectrometry analysis

Sample preparation and resulting mass spectrometry experiments were performed in biological triplicate. BL3 or TBL3 cell pellets were lysed in ice cold lysis buffer by sonication using a Bioruptor (Diagenode) on medium power (200W) for 30 s on/off for twenty cycles, followed by vortexing. The lysis buffer consisted of 8 M urea in 50 mM ammonium bicarbonate (pH 8.0), supplemented with Halt Protease Inhibitor Cocktail (Thermo Fisher Scientific). The lysed samples were centrifuged (10 min, 17,000 x *g*, 4°C). The resulting supernatant was transferred to a fresh tube, reduced (5 mM DTT, 1 h, RT), alkylated (10 mM iodoacetamide, 45 min in dark, RT), and diluted to 1.5 M urea with 50 mM ammonium bicarbonate (pH 8.0).

The samples were digested to peptides using trypsin (Promega) at an enzyme-to-substrate ratio of ∼1:50 (12 h, RT). High pH reverse phase (RP) fractionation was performed on a Hamilton C18 stage tip using 8, 12, 18, 24, 30, 36, 42, 60% acetonitrile in 100 mM ammonium formate buffer (pH 10.0) to bin peptides by hydrophobicity [61]. Fractions were combined to obtain four final fractions (8% with 30%, 12% with 36%, 18% with 42%, and 24% with 60%). Samples were desalted using P200 columns with C18 3 M plug (3M Bioanalytical Technologies) and reconstituted in 0.1% trifluoroacetic acid (TFA).). Liquid Chromatography–Tandem Mass Spectrometry (LC-MS/MS) was performed with a Thermo Fisher Ultimate 3000 Dionex liquid chromatography system and a Thermo Fusion mass spectrometer. Mobile phase A contained 0.1% formic acid and mobile phase B contained 0.1% formic acid and 80% acetonitrile. Peptides were separated at a flow rate of 300 nl/min along a 120 min gradient from 2-50% mobile phase B. Samples were quantified by A280 absorbance and 1 µg of each was injected onto a laser pulled 2.4 µm C18 particle size silica column of 35 cm in length. Samples were run with MS1 settings of 300–1200 m/z window, a resolution of 60,000, normalized AGC target of 125%, and maximum inject time (MIT) of 54 ms. Fragment (MS2) scans were collected in data dependent mode with a TopN loop count of 10, resolution of 30,000, normalized AGC target of 150%, and MIT of 100 ms. Fragmentation was performed with HCD using normalized collision energies (NCE) of 29%.

The raw mass spectrometry files were searched for protein identification and quantification using the Proteome Discoverer software suite (v2.4, Thermo Scientific) implementing the Sequest HT algorithm (10 ppm tolerance for MS1, 0.02 Da tolerance for MS2). The fractions were merged in the search as a single sample, providing a unique protein abundance quantification within each biological replicate, without explicit identification of the specific fraction that contained the respective peptide. Quantified peptides were searched against the UniProtKB/Swiss-Prot database of *Bos taurus* and *Theileria annulata* [62]. Peptide spectral matches were evaluated using Percolator with an FDR cut off 1%. Protein IDs were selected with a 1% FDR and with peptides less than a q-value of 0.01 [63]. The Minora feature detector (3 ppm tolerance, max retention time of 2 min) was used to match features between runs.

### Sample preparation for CUT&RUN analysis

Buffers were prepared as follows: (i) Binding Buffer: 20 mM HEPES-KOH (pH 7.9), 10 mM KCl, 1 mM CaCl_2_, and 1 mM MnCl_2_; (ii) Wash Buffer: 20 mM HEPES-KOH (pH 7.9), 150 mM NaCl, 0.5 mM spermidine, and EDTA-free protease inhibitors; (iii) Dig-Wash Buffer: Wash Buffer supplemented with 0.05% digitonin; (iv) Antibody Buffer: Dig-Wash Buffer supplemented with 2 mM EDTA; and (v) 2x STOP Buffer: 340 mM NaCl, 20 mM EDTA, 4 mM EGTA, 0.05% digitonin, 0.05 mg/mL RNase A, 0.05 mg/mL GlycoBlue, and 10 pg spike-in DNA per sample.

CUT&RUN was performed using a digitonin-based protocol. Briefly, 5 × 10^5 cells per sample were washed once in Wash Buffer and resuspended in 1 mL Wash Buffer on ice. Concanavalin A-coated magnetic beads were activated by incubation in Binding Buffer for 2 min at 4°C, washed twice with Binding Buffer, and resuspended in the original bead volume. Cells were incubated with 20 µL activated beads for 5 min at room temperature with rotation.

Bead-bound cells were collected on a magnetic rack and incubated with primary antibody diluted 1:50 in Antibody Buffer for 1 h at room temperature with rotation. Samples were washed twice with Dig-Wash Buffer and subsequently incubated with pA-MNase (1:2000 dilution) in Dig-Wash Buffer for 15 min at room temperature. After two additional washes in Dig-Wash Buffer, beads were resuspended in 150 µL Dig-Wash Buffer and chilled for 5 min in an ice-water slurry. Targeted digestion was initiated by addition of 3 µL 100 mM CaCl_2_ and allowed to proceed for 30 min at 0°C. The reaction was terminated by addition of 150 µL 2 × STOP Buffer.

CUT&RUN fragments were released by incubation at 37°C for 20 min with shaking. Samples were centrifuged at 16,000 × g for 5 min at 4°C, placed on a magnetic rack, and the supernatants were transferred to low-binding tubes. DNA was purified by Proteinase K/SDS digestion at 70°C for 30 min, followed by phenol–chloroform extraction and ethanol precipitation using GlycoBlue as carrier. DNA pellets were washed once with 85% ethanol, air-dried, and resuspended in 40 µL of 5 mM Tris-HCl (pH 8.0) and 0.1 mM EDTA for library preparation.

Sequencing libraries were prepared from purified CUT&RUN DNA using the KAPA HyperPrep Kit (Roche) and xGen UDI-UMI adapters (Integrated DNA Technologies) according to the manufacturers’ instructions. Libraries were amplified, purified, and quality-controlled using a TapeStation system prior to sequencing. High-throughput sequencing was performed by the GENOM’IC Platform at Institut Cochin (Paris, France).

### Mass spectrometry data analysis

Potential contaminants and reverse protein sequences were removed from the data. All protein group “0” abundances were converted to “NA” to indicate non-identification of a protein, and the data were filtered to remove any protein groups for which there were no quantifications for any biological replicates. All abundances were transformed to log2 values and median-normalization was performed within each biological replicate. The mean normalized log2 abundance was calculated across the 3 biological replicates of each cell type. The log2 fold change in protein abundance was calculated by comparing the mean normalized log2 abundances for TBL3 cells compared to BL3. To calculate a log2 fold change for a protein, it was required that the respective protein be quantified in at least one biological replicate from each infection condition. A two-tailed, unpaired, students t-test, with the assumption of equal variance, was performed to compare the normalized log2 intensities and test the statistical significance of the differences of the mean protein abundances.

## Supporting information

S1 Table Gene locus information of investigated *Bos taurus* immune gene families. Information was extracted from the GTF annotation file used for feature counting.
(PDF)

S2 Table List of all oligonucleotides used in this study for RT-qPCR analysis. (PDF)

S1 Fig Flow cytometry gating strategy and raw data Alexa Fluor 568. BL3 and TBL3 stained with secondary antibody Alexa Fluor 568, Goat anti-Rabbit IgG (H + L) used as negative controls.
(PDF)

S2 Fig Flow cytometry gating strategy and raw data MHC I ILA88. BL3 stained with primary antibody MHC I ILA88 (recognizing BOLA1) and secondary antibody Alexa Fluor 568, Goat anti-Rabbit IgG (H + L).
(PDF)

S3 Fig Flow cytometry gating strategy and raw data MHC I ILA88. TBL3 stained with primary antibody MHC I ILA88 (recognizing BOLA1) and secondary antibody Alexa Fluor 568, Goat anti-Rabbit IgG (H + L).
(PDF)

S4 Fig Flow cytometry gating strategy and raw data MHC II. BL3 stained with primary antibody MHC II (recognizing BOLA-DQ) and secondary antibody Alexa Fluor 568, Goat anti-Rabbit IgG (H + L).
(PDF)

S5 Fig Flow cytometry gating strategy and raw data MHC II. TBL3 stained with primary antibody MHC II (recognizing BOLA-DQ) and secondary antibody Alexa Fluor 568, Goat anti-Rabbit IgG (H + L).
(PDF)

S6 Fig PCA analysis of transcriptome datasets. (PDF)

S1 Raw Gel Original uncropped and unadjusted images underlying all blot results reported in a submissions figure 4H. (PDF)
