# Supplementary material for: Epigenetic silencing of innate and adaptive immunity genes underlies the immune evasion cancer hallmark in Theileria-infected B-cells
Source: PLoS Pathog. 2026 Aug 28;22(8):e1014498. doi: 10.1371/journal.ppat.1014498 (PMC13600614; doi:10.1371/journal.ppat.1014498)
Supplement: Supplementary file 1 [file ppat.1014498.s001.pdf]

S1 Table

Gene locus information of investigated *Bos taurus* immune gene families.

Information was extracted from the GTF annotation file used for feature counting.

Chromosome #	Chr_Accession	Start	End	Gene Symbol	Protein	Strand
23	NC_037350.1	7263692	7269361	BOLA-DMA	BOLA-DMA	-
23	NC_037350.1	7246589	7253434	BOLA-DMB	BOLA-DMB	-
23	NC_037350.1	7314756	7323452	BOLA-DOA	BOLA-DOA	-
23	NC_037350.1	7114327	7122816	BOLA-DOB	BOLA-DOB	-
23	NC_037350.1	25582894	25589220	BOLA-DQA2	BOLA-DQA2	-
23	NC_037350.1	25631992	25643886	BOLA-DQA5	BOLA-DQA5	-
23	NC_037350.1	25607490	25620857	BOLA-DQB	BOLA-DQB	+
23	NC_037350.1	25838390	25843092	BOLA-DRA	BOLA-DRA	-
23	NC_037350.1	25795926	25828806	BOLA-DRB2	BOLA-DRB2	+
23	NC_037350.1	25723690	25734819	BOLA-DRB3	BOLA-DRB3	+
23	NC_037350.1	7072087	7077975	BOLA-DYA	BOLA-DYA	+
23	NC_037350.1	7096987	7113266	BOLA-DYB	BOLA-DYB	-
7	NC_037334.1	61747920	61755746	CD74	CD74	-
25	NC_037352.1	9542188	9601544	CIITA	CIITA	+
3	NC_037330.1	54186301	54507575	GBP4	GBP4	+
3	NC_037330.1	54094966	54111588	GBP5	GBP5	+
3	NC_037330.1	54043363	54062086	GBP6	GBP6	+
3	NC_037330.1	54342869	54349809	LOC112445995	GBP7-like	+
3	NC_037330.1	54715941	54782317	LOC507055	GBP4	+
3	NC_037330.1	54716017	54921064	LOC511531	GBP1	+
3	NC_037330.1	54148958	54165067	LOC512486	GBP1	-
3	NC_037330.1	53862383	53891399	LOC513659	GBP6	+
3	NC_037330.1	54409420	54433493	LOC781710	GBP2	+
6	NC_037333.1	58034096	58041090	TLR10	TLR10	-
17	NC_037344.1	3953806	3990089	TLR2	TLR2	-
27	NC_037354.1	16176312	16187575	TLR3	TLR3	+
8	NC_037335.1	107057825	107068842	TLR4	TLR4	+
6	NC_037333.1	58050660	58091639	TLR6	TLR6	-
X	NC_037357.1	130768541	130787093	TLR7	TLR7	-
X	NC_037357.1	130728234	130745827	TLR8	TLR8	-
22	NC_037349.1	48676669	48680933	TLR9	TLR9	+
