# Supplementary material for: Epigenetic silencing of innate and adaptive immunity genes underlies the immune evasion cancer hallmark in Theileria-infected B-cells
Source: PLoS Pathog. 2026 Aug 28;22(8):e1014498. doi: 10.1371/journal.ppat.1014498 (PMC13600614; doi:10.1371/journal.ppat.1014498)
Supplement: Supplementary file 2 [file ppat.1014498.s002.pdf]

S2 Table

List of all oligonucleotides used in this study for RT-qPCR analysis.

Gene	Forward sequence	Reverse sequence
BOLA1	TGCCATTCGCACGAAGTTG	GACTTTTCTTGCTCCCACCC
BOLA-DMA	CTGCTCTCCTTTCTGTGGCT	CACTCGGATGCTCTGGGAAA
BOLA-DMB	TGCAGGTAGCCAAAACCACT	CCACGTCCGCTGGATAGAAG
BOLA-DOA	GCCTTCTACCAGTCCTATGATG	TCACCAAGTCCTCCAGATG
BOLA-DOB	TTCACCAATGGGACAGAAAAG	CGTCAAGGCCACAAACATC
BOLA-DRA	TCCTGTCTGTTCTAATCCACC	CTTTAATAGCCCACGATTCCTG
BOLA-DRB3	TGTCATTTCTTCAACGGGACC	CGCACGTACTCTTCTCCATTATG
CD74	CATGGATGGCTTGGACTGGA	ATAGTTGCCGTTCTCGTCGT
CIITA	CACTGTCCACTGCCTTGGG	GTAGGGAGACTGGCCAGAGA
HSP70	ACGCAAATGGAATCCTCAAC	TATTCGTCGTGCTCTGCTAA
H2A	GTCGTGGCAAGCAAGGAG	GATCCGGCCGTTAGGTACTC
GBP1	CCCAGCTAGATAAAAAGCGAG	CAATGAAGAGGCAATAGCCC
GBP4	ACTATGACCAGCAGATGGAC	GACTTCAATGGCCTCTTTCTC
GBP5	TTTAATCTGCCCCGTCTGTG	TGGAATGGCTAAAGATGTAGGAAC
TLR4	AGTTAAAGGATGTTCATCTTCCAG	TGCAGCATCCTGAGATACCAGG
TLR6	TCCTGCCATCCAATCACCAC	GTCAAGTTGCCAAATTCCTTACA
TLR7	CTGTGATGTCACTCTGGATG	TAAGGTTGGTGGCATTGGCA
TLR10	AACTTCTCCAGCTTCCCAGAACTGC	GGAGAGGTGGTTTAGGCCCTGA
