# Supplementary material for: Epigenetic silencing of innate and adaptive immunity genes underlies the immune evasion cancer hallmark in Theileria-infected B-cells
Source: PLoS Pathog. 2026 Aug 28;22(8):e1014498. doi: 10.1371/journal.ppat.1014498 (PMC13600614; doi:10.1371/journal.ppat.1014498)
Supplement: Supplementary file 3 [file ppat.1014498.s003.pdf]

S1 Fig

BL3 and TBL3 stained with secondary antibody Alexa Fluor™ 568, Goat anti-Rabbit IgG (H+L) used as negative controls.

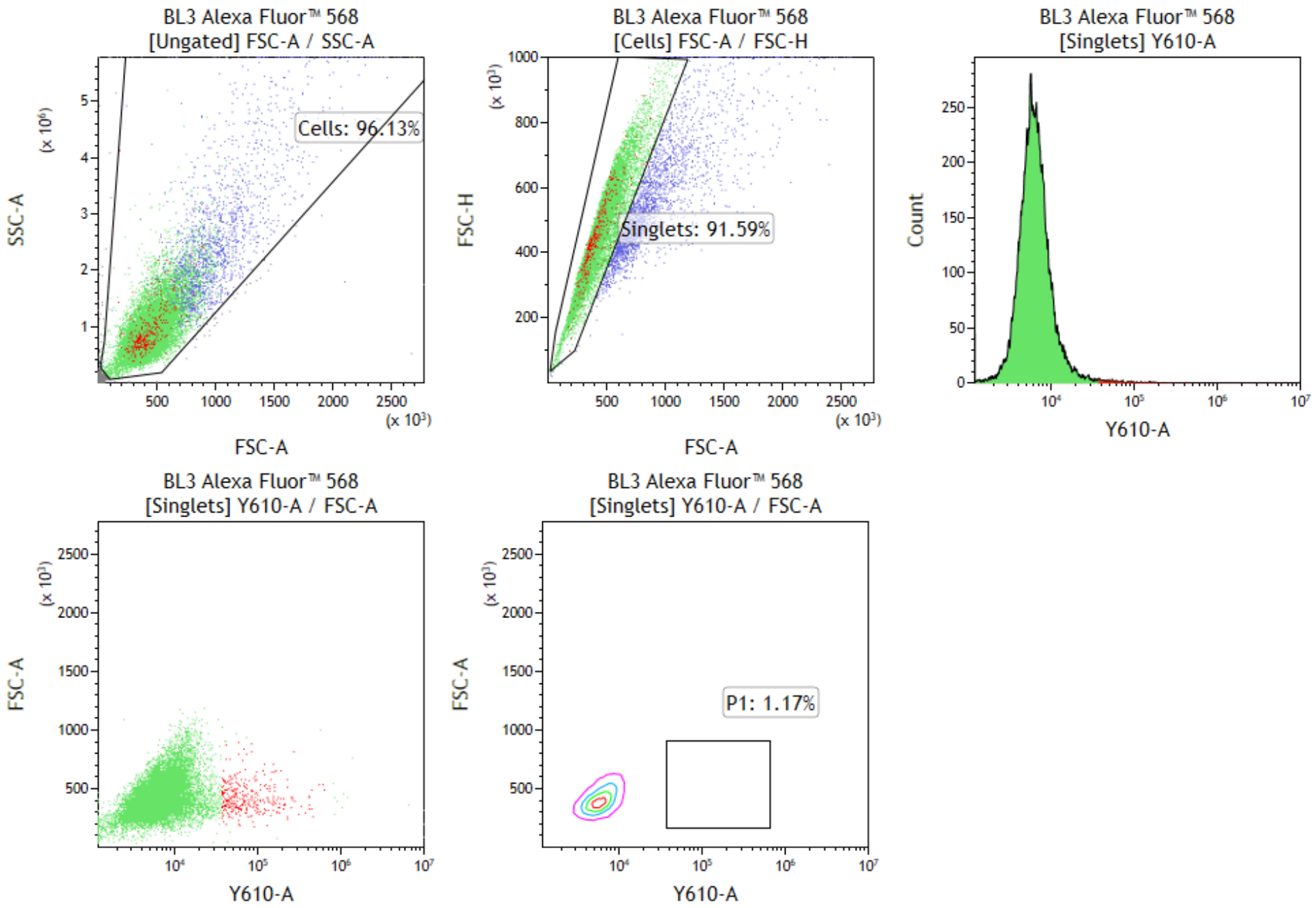

BL3 Alexa Fluor™ 568

Gate	Number	%Total	%Gated	"FL9-A"-GMean-Log	Logic
All	30,000	100.00	100.00	6262.90	Ungated
Cells	28,839	96.13	96.13	6957.02	Cells
Singlets	26,415	88.05	91.59	6445.78	Singlets AND Cells
P1	308	1.03	1.17	76414.66	P1 AND Singlets AND Cells

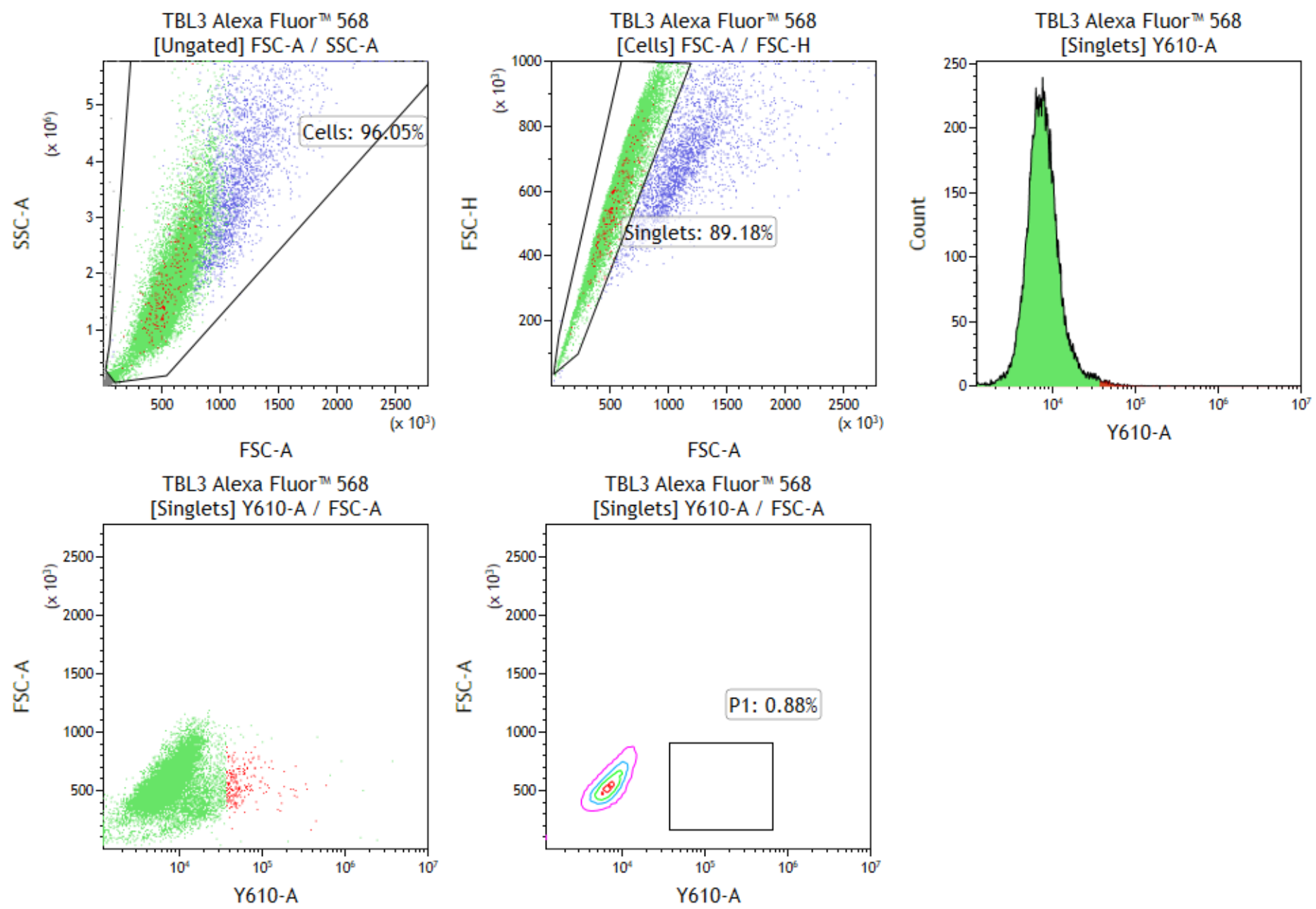

TBL3 Alexa Fluor™ 568

Gate	Number	%Total	%Gated	"FL9-A"-GMean-Log	Logic
All	30,000	100.00	100.00	7416.27	Ungated
Cells	28,815	96.05	96.05	8262.91	Cells
Singlets	25,698	85.66	89.18	7523.50	Singlets AND Cells
P1	226	0.75	0.88	58380.99	P1 AND Singlets AND Cells
