# Supplementary material for: Epigenetic silencing of innate and adaptive immunity genes underlies the immune evasion cancer hallmark in Theileria-infected B-cells
Source: PLoS Pathog. 2026 Aug 28;22(8):e1014498. doi: 10.1371/journal.ppat.1014498 (PMC13600614; doi:10.1371/journal.ppat.1014498)
Supplement: Supplementary file 7 [file ppat.1014498.s007.pdf]

S5 Fig

TBL3 stained with primary antibody MHC II (recognizing BOLA-DQ/BOLA-DR) and secondary antibody Alexa Fluor™ 568, Goat anti-Rabbit IgG (H+L).

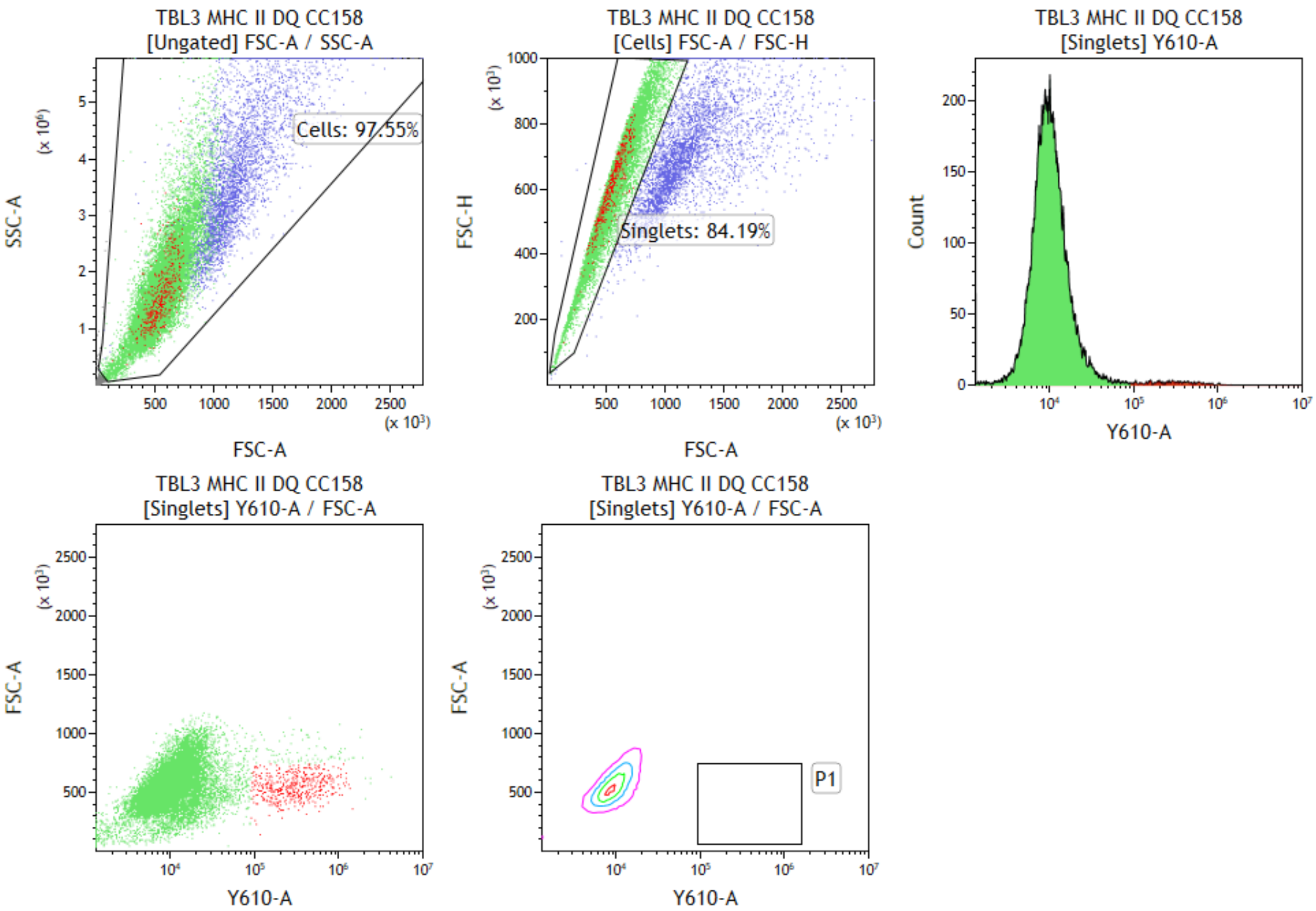

TBL3 MHC II DQ CC158

Gate	Number	%Total	%Gated	"FL9-A"-GMean-Log	Logic
All	30,000	100.00	100.00	11719.95	Ungated
Cells	29,266	97.55	97.55	12322.21	Cells
Singlets	24,640	82.13	84.19	10632.60	Singlets AND Cells
P1	458	1.53	1.86	289773.34	P1 AND Singlets AND Cells
