# Supplementary material for: Epigenetic silencing of innate and adaptive immunity genes underlies the immune evasion cancer hallmark in Theileria-infected B-cells
Source: PLoS Pathog. 2026 Aug 28;22(8):e1014498. doi: 10.1371/journal.ppat.1014498 (PMC13600614; doi:10.1371/journal.ppat.1014498)
Supplement: Supplementary file 8 [file ppat.1014498.s008.pdf]

S6 Fig

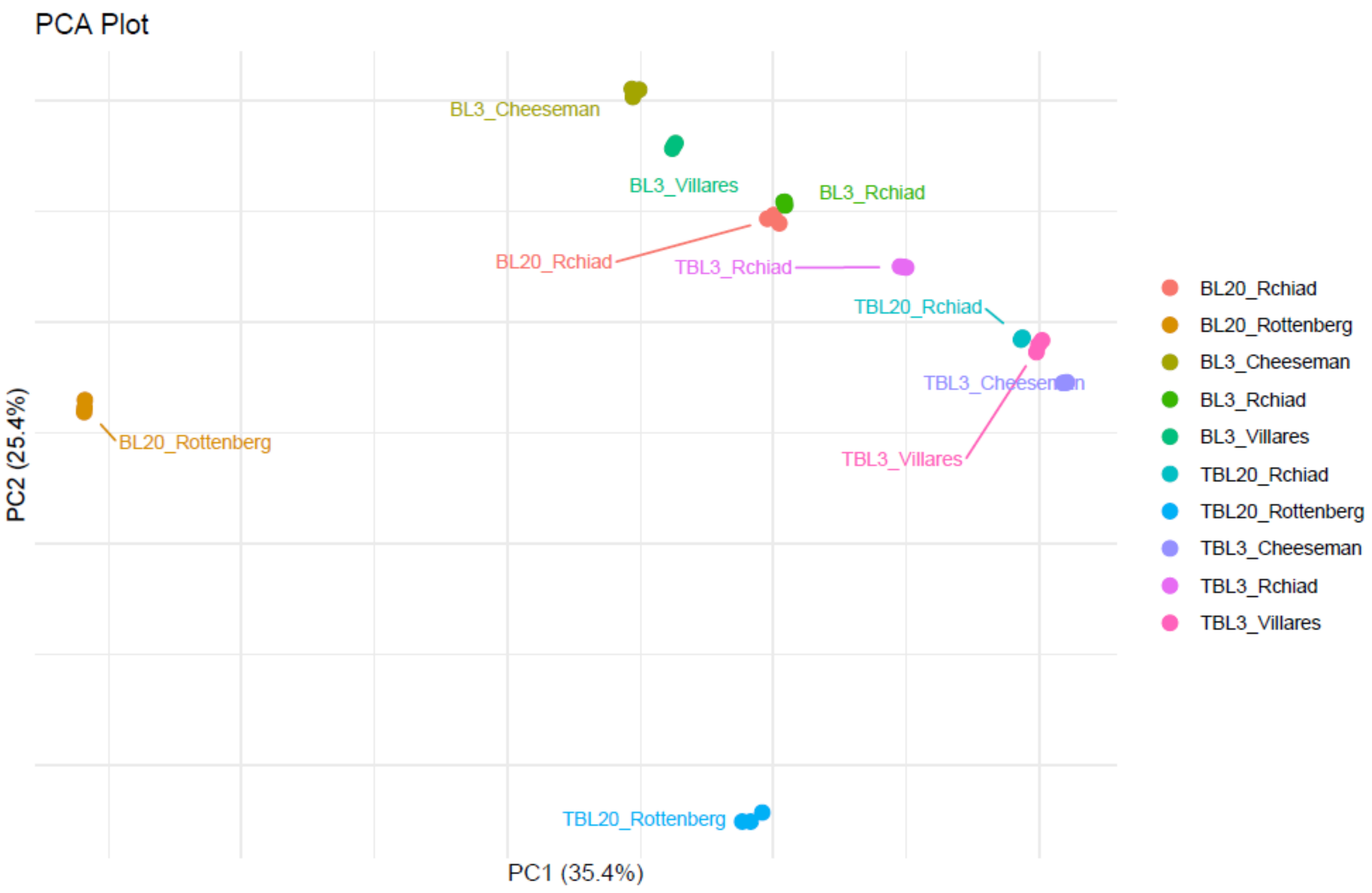
PCA Plot
BL3_Cheeseman
BL3_Villares
BL3_Rchiad
BL20_Rchiad
TBL3_Rchiad
TBL20_Rchiad
TBL3_Cheeseman
TBL3_Villares
BL20_Rottenberg
TBL20_Rottenberg
PC2 (25.4%)
PC1 (35.4%)
BL20_Rchiad
BL20_Rottenberg
BL3_Cheeseman
BL3_Rchiad
BL3_Villares
TBL20_Rchiad
TBL20_Rottenberg
TBL3_Cheeseman
TBL3_Rchiad
TBL3_Villares
